# Lipreading a naturalistic narrative in a female population: Neural characteristics shared with listening and reading

**DOI:** 10.1002/brb3.2869

**Published:** 2022-12-29

**Authors:** Satu Saalasti, Jussi Alho, Juha M. Lahnakoski, Mareike Bacha‐Trams, Enrico Glerean, Iiro P. Jääskeläinen, Uri Hasson, Mikko Sams

**Affiliations:** ^1^ Department of Psychology and Logopedics University of Helsinki Helsinki Finland; ^2^ Brain and Mind Laboratory, Department of Neuroscience and Biomedical Engineering Aalto University Espoo Finland; ^3^ Advanced Magnetic Imaging (AMI) Centre, Aalto NeuroImaging, School of Science Aalto University Espoo Finland; ^4^ Independent Max Planck Research Group for Social Neuroscience Max Planck Institute of Psychiatry Munich Germany; ^5^ Institute of Neuroscience and Medicine, Brain & Behaviour (INM‐7) Research Center Jülich Jülich Germany; ^6^ Institute of Systems Neuroscience Medical Faculty Heinrich Heine University Düsseldorf Düsseldorf Germany; ^7^ Department of Psychology and the Neuroscience Institute Princeton University Princeton USA; ^8^ Department of Neuroscience and Biomedical Engineering Aalto University Espoo Finland; ^9^ Aalto Studios ‐ MAGICS Aalto University Espoo Finland

**Keywords:** fMRI, language, lipreading, speech, speechreading

## Abstract

**Introduction:**

Few of us are skilled lipreaders while most struggle with the task. Neural substrates that enable comprehension of connected natural speech via lipreading are not yet well understood.

**Methods:**

We used a data‐driven approach to identify brain areas underlying the lipreading of an 8‐min narrative with participants whose lipreading skills varied extensively (range 6–100%, mean = 50.7%). The participants also listened to and read the same narrative. The similarity between individual participants’ brain activity during the whole narrative, within and between conditions, was estimated by a voxel‐wise comparison of the Blood Oxygenation Level Dependent (BOLD) signal time courses.

**Results:**

Inter‐subject correlation (ISC) of the time courses revealed that lipreading, listening to, and reading the narrative were largely supported by the same brain areas in the temporal, parietal and frontal cortices, precuneus, and cerebellum. Additionally, listening to and reading connected naturalistic speech particularly activated higher‐level linguistic processing in the parietal and frontal cortices more consistently than lipreading, probably paralleling the limited understanding obtained via lip‐reading. Importantly, higher lipreading test score and subjective estimate of comprehension of the lipread narrative was associated with activity in the superior and middle temporal cortex.

**Conclusions:**

Our new data illustrates that findings from prior studies using well‐controlled repetitive speech stimuli and stimulus‐driven data analyses are also valid for naturalistic connected speech. Our results might suggest an efficient use of brain areas dealing with phonological processing in skilled lipreaders.

## Graphical abstract

Female participants lipread, listened to, andread the same narrative. We analysed the similarity of brain activity with within‐and between‐conditioninter‐subject correlations. We also analysed which brain areas were related tolipreading test score and experienced comprehension of the lipread narrative.

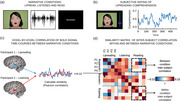

## 1 INTRODUCTION

To some extent, everyone can extract phonetic information from a speaker's lips, tongue, jaw, teeth, and cheeks, and use this ability, known as lipreading or speechreading, to support speech comprehension (Bernstein & Liebenthal, 2014; Files et al., 2015). This is particularly useful for individuals with hearing impairment (Strelnikov et al., 2009; Suess et al., 2022) even after cochlear implantation (Anderson, Wiggins, Kitterick, & Hartley, 2017), but also for normal hearing individuals when speech is difficult to comprehend, for example, due to acoustic noise (Ross et al., 2007; Sumby & Pollack, 1954). For most normal hearing individuals, comprehending speech solely via lipreading is challenging, due to poor visibility of some sounds (e.g., the velopharyngeal sounds /k/ and /h/), and due to the similarity between the lip shapes of different phonemes (e.g., the bilabial phonemes /m/, /b/, and /p/) (Altieri et al., 2011; Auer & Bernstein, 2007; Bernstein & Liebenthal, 2014; Files et al., 2015; Summerfield et al., 1992). Consequently, lipreading accuracy varies greatly (from 0% to 65%, mean 18.57%; SD = 13.18) in individuals with normal hearing (Altieri et al., 2011), and the benefits of good lipreading are not available in the same way for all. A better understanding of the neural basis of lipreading comprehension, especially the comprehension of naturalistic speech is critical for understanding how the brain accesses linguistic meaning via different means. This will help in developing more efficient intervention methods and allow more people to access the benefits of fluent lipreading.

In order to comprehend speech via lipreading, visual speech movements need to be recognized by mapping sensory or phonological representations to their meanings, similarly to the manner in which familiar speech sounds are recognized in auditory speech (DeWitt & Rauschecker, 2012; Hickok & Poeppel, 2007), and familiar orthographic forms in text comprehension (Dehaene & Cohen, 2011). Different theories postulate possible visual speech processing pathways. One theory suggests convergence with auditory speech pathways, which is supported by findings showing that viewing naturally moving speaking faces versus still images of a resting face activates auditory speech areas (BA 41/42; lateral parts of Heschl's gyrus, Calvert & Campbell, 2003). Similarly, lipreading meaningless syllables (Möttönen et al., 2002; Pekkola et al., 2005; Sams et al., 1991) and numbers (Calvert et al., 1997; MacSweeney et al., 2000) as well as words (Calvert et. al., 1997) has been found to activate the auditory cortex, including its primary areas. Alternatively, it has also been suggested that the phonetic integration of visual speech cues would be processed bottom‐up within the late vision system (Bernstein et al., 2004; Bernstein et al., 2011; Paulesu, 2003). Potentially both auditory and visual cortices have a functionally specific form, and brain engages both visual and auditory pathways for comprehension (Bröhl et al., 2022). Or visual speech processing would be based on multisensory integration in the superior temporal lobe (Okada et al., 2013; Beuchamp et al., 2004; Calvert et al., 2000). Furthermore, participation of the prefrontal speech motor areas in the middle frontal gyrus (MFG), inferior frontal gyrus (IFG), supramarginal gyrus (SMG), and premotor cortex has been found when viewing orofacial movements (Nishitani & Hari, 2002), indicating the involvement of a mirror neuron‐like system. The speech motor system was activated when participants attempted to lipread vowels (Callan et al., 2014), syllables (Skipper et al., 2007), words (Paulesu et al., 2003; Watkins et al., 2003), and short stories (Skipper et al., 2005). More recently, it has been suggested that a specific temporal visual speech area (TVSA), in the posterior temporal cortex, ventral and posterior to the multisensory posterior superior temporal sulcus (pSTS), supports sensory–motor integration (Bernstein & Liebenthal, 2014; Bernstein et al., 2011), because its strength of activation has been found to be related to the number of correctly recognized nonsense syllables (*n* = 10). But further brain areas have been found to covary with lipreading accuracy measures.

Capek et al. (2008) found lipreading a skill‐dependent activity in the right lingual gyrus, the posterior cingulate (pCING), and in the right postcentral and inferior temporal gyri in normal hearing individuals (*N* = 13). The authors suggested that this was due to the relatively greater involvement of face processing and articulatory skill. Furthermore, an early functional magnetic resonance imaging (fMRI) study, based on data from nine participants, suggested that poor versus good lipreaders had less activation in the superior temporal gyrus (STG) and middle temporal gyrus (MTG) while lipreading sentences (Ludman et al., 2000). In a more comprehensive study, 33 normally hearing participants with lipreading skills ranging from 7% to 89% (quantified with a sentence‐based lipreading test) watched silent, naturally spoken, isolated sentences during fMRI (Hall et al., 2005). Irrespective of lipreading skills, activity was found in the IFG, MFG, inferior parietal lobule (IPL), particularly in the left hemisphere, and MTG (peak activity in the posterior part) bilaterally. A correlation analysis suggested that small voxel clusters (4−11 voxels) in the mSFG, IFG, fusiform gyrus, pCING cortex, and lingual gyrus (bilaterally) were associated with lipreading skills. Additionally, the authors found that the activity strength in the left STG showed a significant linear relationship with lipreading skills when using individually thresholded activation maps and restricting the analysis to the STG (the outermost anatomical boundaries of Heschl's gyrus and the planum temporale). The authors suggested that phonological processing mechanisms were important for successful lipreading.

Previous findings suggest that lipreading sublexical material such as pseudowords and syllables elicits activation that is more restricted to areas sensitive to visual motion (Bernstein et al., 2011; Campbell et al., 2001). Instead, lipreading words (Paulesu et al., 2003) and sentences (Hall et al., 2005; Ludman et al., 2000) has been found to elicit activation that extends to the temporal cortex, including MTG in skilled lipreaders. The involvement of MTG increases with the linguistic complexity of the lipread material (Ludman et al., 2000; Paulesu et al., 2003). That said the brain areas that are specifically important for lipreading naturalistic, connected speech are not yet properly identified. Working with naturalistic narratives has become increasingly common (Simony et al., 2016; Wilson et al., 2008; Yeshurun et al., 2017a; for a review, see Jääskeläinen et al., 2021, and for related M/EEG research Crosse et al., 2016; Golumbic et al., 2013; Haider et al., 2022). Listening to naturalistic narratives synchronizes brain activation in higher‐level brain functions reflecting semantic and socio‐emotional processing, for example (Lerner, Honey, Silbert, & Hasson, 2011; Nastase, Gazzola, Hasson, & Keysers, 2019; Regev et al., 2013; Wilson et al., 2008). Interestingly, Regev et al. (2013) found that the neural responses were both selective and invariant to spoken and written narratives. Here we address two research questions. First, to what extent lipreading naturalistic, connected speech engages neural mechanisms underlying lower‐ and higher‐level psycholinguistic processing similar to listening and reading? Second, which brain areas are most relevant in lipreading connected speech?

**FIGURE 1:**
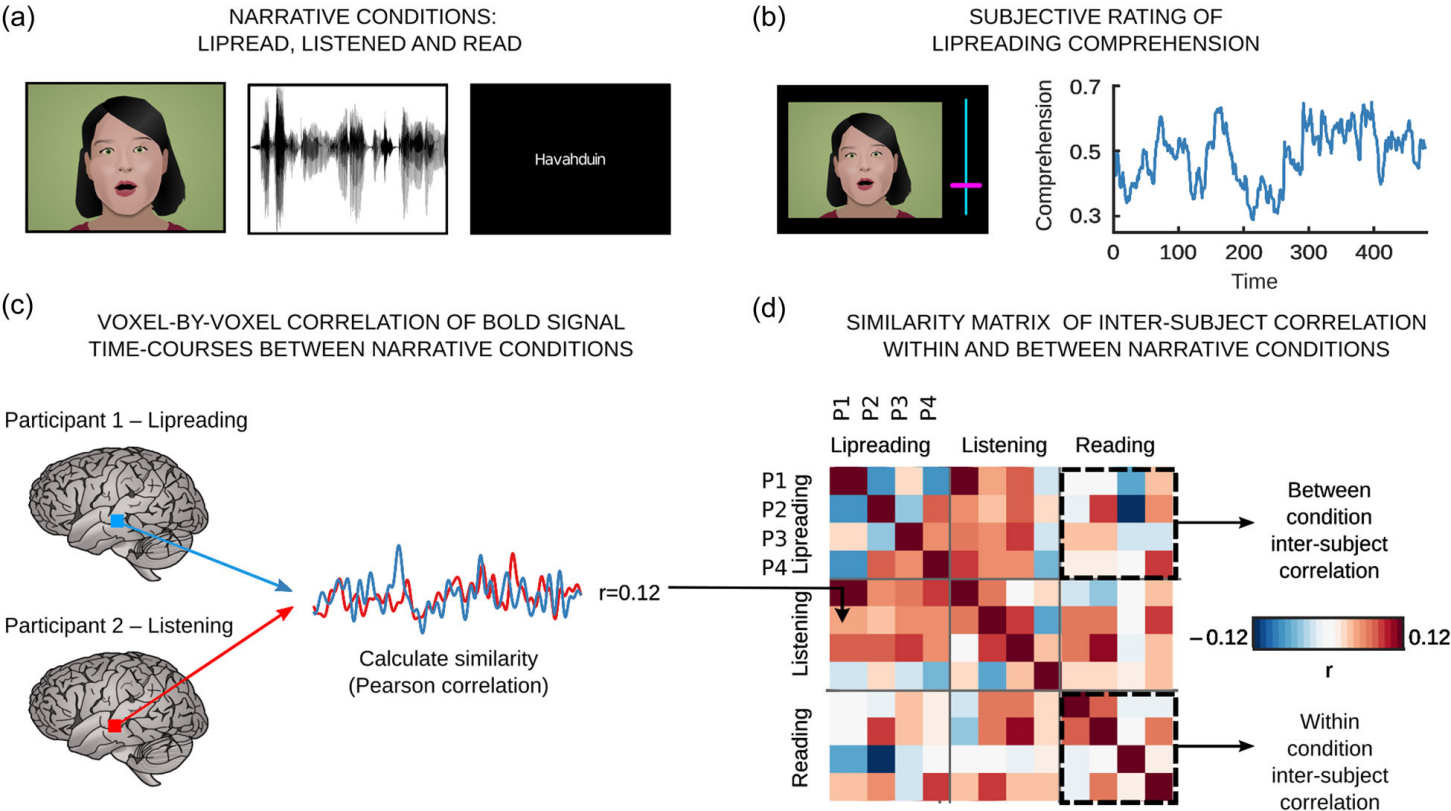
Illustration of the experimental design and between‐narrative‐type inter‐subject correlation (ISC) (A cartoon face is used to conceal the identity of the speaker.) (a) The participants lipread, listened to, and read the same narrative. (b) After scanning, the participants lipread the narrative again and rated how well they comprehended what the speaker said. (c) We used the BOLD signal time courses of one participant during listening as a model for the activity time course of other participants in the same voxel to identify brain areas with similar time courses during lipreading: we computed the ***r*** statistics voxel‐by‐voxel between the signals and repeated the process for all participant pairs. (d) The similarity matrix of four participants depicting between‐ and within‐condition inter‐subject correlations from one voxel in the posterior temporal cortex. In contrast to previous studies, we analyzed the between‐condition ISCs

In this study, our aim was (1) to identify cortical processing areas underlying lipreading naturalistic, connected speech, and (2) to evaluate to what extent the higher‐level brain areas of listening and reading comprehension are recruited by lipreading the same narrative. Furthermore, we aimed (3) to identify brain areas where activity is predicted by better comprehension of lipread connected speech. To this end, participants with large inter‐individual variation in lipreading skills had their brain activity measured with 3‐T fMRI while they lipread an 8‐min narrative from a silent video showing the speaker's face, listened to the same narrative without seeing the face of the speaker, and read a time‐locked transcript of the narrative (Figure 1).

We hypothesized that lipreading a naturalistic narrative would involve cortical areas in the occipital, temporal, and parietal cortices. In addition to these low‐level speech processing areas, we hypothesized that cortical areas responsible for lexical–semantic and higher‐level linguistic processing in the parietal and frontal cortices would be involved, similarly to the way they are involved during listening to and reading the narrative. However, depending on how well the narrative is comprehended via lipreading, we expected that the activation in the latter areas might be spatially more restricted during lipreading than during listening to and reading the narrative. Finally, we hypothesized that better comprehension of the lipread narrative would be enabled by the same cortical processing areas in the temporal cortex that are used in coding heard speech.

## 2 METHODS

### 2.1 Participants

The volunteer participants were 31 healthy native Finnish‐speaking females (mean age 30.9 years, range 20−49), all reported normal hearing. The narrative was related from the female first‐person perspective to maximize the engagement of the participants with the narrative. There is also evidence suggesting that women and men may use different neural mechanisms, because females had more activity in the left auditory area while lipreading silently articulated numbers, although their number recognition accuracy was similar to males (Ruytjens et al., 2006; Ruytjens et al., 2007). Thus, to focus on individual differences in lipreading skills, we wanted to exclude between‐sex variability in the current study. One participant's data was removed due to excessive head movement, and another's due to poor attention (eyes closed during scanning for approximately 3 min), resulting in a final sample of 29 participants. All participants were right handed (Edinburgh handedness inventory, Oldfield, 1971), and reported normal hearing and normal or corrected to normal (with contact lenses) vision, and no psychiatric or neurological disabilities. All participants signed informed consent forms, and received monetary compensation for their time. The study was approved by the research ethics committee of Aalto University and was conducted in accordance with the Helsinki Declaration for Human Studies.

We developed an online test for screening lipreading skills. We first recorded a female speaker with clear visual articulation, speaking 100 sentences that were translated into Finnish from the CID everyday sentences with varying length and sentence structure (Sims, 1975). Out of 100 sentences, 10 sentences that mimic the sentence structure of the Finnish language well were chosen for an online screening tool for participant recruitment. The online screening tool was distributed with information on participant requirements via the student mailing lists of local academies including speech therapy and sign language interpreters, and via the Finnish Federation of Hard of Hearing to reach individuals with higher lipreading skill but normal hearing. The participants were instructed to type out the words they recognized from the silent videos. The number of correctly recognized words out of a total of 57 words was used as each participant's lipreading skill score. For the current study, we then chose individuals with a large inter‐individual variation in their lipreading skills, which were again tested on site with another test (Lonka, 1993).

### 2.2 Stimuli and experimental design

The stimulus was a narrative (duration 7 min 54 s) related by a female speaker, portraying the events and thoughts that occurred during her day from a first‐person perspective. The narrative conditions were: audio only (listening), visual only (lipreading a silent video of a speaking face), and text only (reading). The study design also contained an additional dimension: an unintelligible, gibberish version of the same narrative was presented to the participants as audio, visual, and text, so that the full experimental design consisted of six narrative conditions altogether. The gibberish was created by replacing consonants from each word of the original narrative with other consonants with a similar place of articulation, but the suffixes that indicated syntax remained unchanged. This resulted in a meaningless string of speech sounds that had very similar facial gestures and acoustic properties and structure (syntax) to the original narrative, but no content (semantics), sounding phonetically natural. The gibberish narratives always preceded the corresponding intact narratives, which were then presented in an order counterbalanced across the participants (Figure 1a). The full results related to the gibberish narrative will be reported separately. The speaker, who was chosen for her clear visual articulatory gestures, was video‐recorded reading the narrative aloud with neutral prosody from a prompter in an acoustically shielded room using an additional Sennheiser EW 112P‐G3‐C microphone and a Canon XA10 video camera. The speaker had rehearsed reading the stories aloud at home, and the high phoneme–grapheme correspondence of the Finnish language eased the natural reading of the gibberish. Two external LED lights (Dyna‐Core Elf2‐DS LED) illuminated the speaker's face against a pale green background canvas to provide good visibility of articulatory movements.

The stimuli were edited with Matlab (MathWorks Inc.). The playback speed of the audio time course was dynamically adjusted to make the narrative types as similar as possible. Each paragraph's starting points were marked manually and the timing of the narrative audio tracks was matched by stretching the audio waveform to maximize the similarity of the loudness envelopes. Playback speeds were between 95% and 101% of the original speed during the paragraphs to make the changes in playback speed imperceptible to the participants (higher deviations from normal playback speeds were allowed during pauses between paragraphs to align the beginning of each paragraph between modalities). The root mean square (RMS) envelopes of the two stimuli after this transformation were highly correlated (*r* = 0.57 for RMS in 0.5‐s windows; *r* = 0.88 after convolution with a canonical hemodynamic response function [HRF]). A similar action was performed on the video, and it was speeded up and slowed down similarly to the audio track using the same timestamps. The video frames were linearly interpolated according to the variable playback speed and resampled back to the original, constant frame rate. The playback speed was varied based on the manually marked timepoints at the end and beginning of each paragraph, so that the changes were implemented during natural pauses in the narrative.

The video recording was divided into visual and audio files. Silent videos of the speaker's face against a light green background were used in the lipreading condition. In the auditory condition, the narrative was presented with a blank screen in a similar shade of green to the background of the lipreading condition to avoid possible effects of still face image on speech perception (Calvert & Campbell, 2003). The participants’ fixation was not controlled in order to approximate a natural listening condition.

We also created a written 614‐word transcript of the narrative. The written words were presented centrally on the screen time‐locked to each word of the original spoken narrative. When the duration of the words in the spoken narrative was very short, two or three words were presented simultaneously to maintain the timing and ensure the legibility of the text.

Stimuli were presented using Presentation software (Neurobehavioral Systems Inc., Albany, California, USA). The audio stimuli were played with MRI‐compatible in‐ear earbuds (Sensimetrics S14 insert earphones). In addition, MRI‐safe protective earmuffs were placed over the earbuds for noise removal and safety. Sound intensity was adjusted for each participant to be loud enough to be heard over the scanner noise by playing example stimuli that were normalized to the same level as the auditory stories during a dummy EPI sequence before the actual experiment. In the MRI scanner, the stimulus videos and texts were back‐projected onto a semitransparent screen, using a Panasonic PT‐DZ110XEJ projector. The viewing distance was 35 cm, the width and height of the projected face image was 380 pixels in height and 161 pixels in width (video display resolution 650 × 490), corresponding to approximately 10.9° vertical and 4.6° horizontal angle in the visual field.

Each stimulus presentation began with a fixation cross in the middle of the screen.

#### 2.2.1 Assessment of lipreading skills

Prior to scanning, each participant's individual lipreading skills were confirmed on‐site with a sentence‐based lipreading test, comprising 10 sentences of variable length (Lonka, 1993). The test stimuli were presented on a 17″ computer screen (resolution 1366 × 768) with a 40‐cm viewing distance. The face image (height 9.6 cm, width 6 cm) on the screen corresponded to a 12.8° vertical and 8.5° horizontal angle in the visual field. The speaker repeated each of the 10 sentences twice: first by saying each one at a slower‐than‐usual speech rate, and then at a normal speech rate. Participants were instructed to write down the words they were able to recognize. The number of correctly recognized words (as a percentage out of a maximum of 50) provided the lipreading skill score. For example, if the sentence was “On Thursday we eat pancakes” and a participant wrote down “we eat,” she got a score of 2 out of 5.

Immediately after the fMRI experiment, the participants watched the silent visual narrative again, and rated their comprehension (on a continuous scale from very poor to very good). The instruction was to estimate how well they had comprehended the narrative when it was first presented to them in the scanner. The subjective rating was performed after fMRI acquisition to prevent any influence on the neural activity during scanning, and the participants were not aware that they would be asked to rate their comprehension afterwards. The rating was conducted using a web‐based dynamic rating tool (https://version.aalto.fi/gitlab/eglerean/dynamicannotations) (see Nummenmaa et al., 2012). The data were collected at 5 Hz. Participants used a mouse to move a small cursor up (good comprehension) and down (poor comprehension) on the right‐hand side of the screen. The original scale of the rating was from 0 (very poor) to 1 (very good).

#### 2.2.2 MRI acquisition

fMRI was performed with a 3T Magnetom Skyra whole‐body scanner (Siemens Healthcare, Erlangen, Germany) and a standard 20‐channel receiving head/neck coil at the Advanced Magnetic Imaging (AMI) Centre of the Aalto NeuroImaging infrastructure at Aalto University School of Science. For functional scans, images were acquired using a T2‐weighted echo planer imaging (EPI) pulse sequence: repetition time (TR), 1700 ms; echo time (TE), 24 ms; flip angle, 70°, each volume comprising 33 slices of 4 mm thickness with 0 mm gap; in‐plane resolution was 3 × 3 mm^2^ (field of view, 192 × 192 mm^2^). A total of 295 volumes were acquired from which 13 volumes were discarded from each run to exclude brain activity during the viewing of the pre‐stimulus fixation cross and after ending the actual stimulus presentation. Anatomical T1‐weighted structural images were acquired at a resolution of 1 × 1 × 1 mm^3^ (MPRAGE pulse sequence, TR 2530 ms, TE 3.3 ms, TI 1100 ms, flip angle 7°, 256 × 256 matrix, 176 sagittal slices).

To monitor the participants’ attention, their eye gaze was recorded with an EyeLink 1000 eye tracker (SR Research, Mississauga, Ontario, Canada; sampling rate 1000 Hz, spatial accuracy 0.5°). Prior to the experiment, a nine‐point calibration and validation was performed.

### 2.3 Data analysis

#### 2.3.1 Pre‐processing

The fMRI data were pre‐processed with FSL software (www.fmrib.ox.ac.uk/fsl) using the BRAMILA parallel pre‐processing pipeline (https://version.aalto.fi/gitlab/BML/bramila). First, after correcting for slice‐timing during acquisition, the EPI volumes were spatially realigned to the middle scan by rigid body transformations to correct for head movements using FSL MCFLIRT. EPI and structural images were co‐registered and normalized to each individual's anatomical scan (linear transformation with nine degrees of freedom with FSL FLIRT; structural images were cleared from non‐brain tissues with FSL BET) followed by a linear transformation from anatomical to standard MNI template space (12 degrees of freedom; FSL FLIRT). Finally, BOLD time series were detrended (linear detrend), motion parameters were regressed out (24 parameters expansion) as well as average signals at deep white matter, ventricles and cerebro‐spinal fluid (Power et al., 2014). Finally, a temporal high‐pass filter with a cut‐off frequency of 0.01 Hz was applied, followed by spatial smoothing with a Gaussian kernel of 8‐mm FWHM. The anonymized data that support the findings of this study are available on reasonable request from the corresponding author. The data are not publicly available due to restrictions, for example, they contain information that could compromise the privacy of research participants.

#### 2.3.2 Similarity of brain activity between narrative types measured with inter‐subject correlation of BOLD signal time courses within and between narrative conditions

The data were analyzed with voxel‐wise comparison of the BOLD signal time courses triggered by the listened to, read, and lipread narratives. We estimated the similarity of the time series using inter‐subject correlation analysis (ISC; Hasson et al., 2004), examined the temporal similarity of the signals in individual voxels. ISC is a model‐free, data‐driven method, which has been found suitable for more ecologically valid stimulus paradigms (Hasson et al., 2004; Kauppi et al., 2010) optimal for analyzing data acquired from experiments with complex stimuli by quantifying the similarity of BOLD signals of different participants (Hasson et al., 2010; Kauppi et al., 2010; Pajula et al., 2012). The data were analyzed by comparing BOLD time courses over the narrative listening, and the ISC method used one participant's brain activity as a model to predict brain activity within another participant. Correlations were used as a similarity metric between the BOLD signals of a participant pair, and statistical inference was performed by identifying the brain area where the similarities between subject pairs were consistently larger than a null distribution. A recent paper has shown that the stimulus structure can have an effect on ISC (Lu et al., 2016). Therefore, as recommended, we controlled the possible effect of silent pauses by modeling the stimulus structure based on the presence of speech as in Lahnakoski et al. (2012). We used linear regression by fitting a vector with values of one at the time points of silences. The vector was convolved with the canonical HRF to take into account the delay of the BOLD response. The residuals from the linear model were used for subsequent analyses. ISC was calculated using the ISC toolbox (Kauppi et al., 2010). First, ISC matrices were obtained for each brain voxel by calculating all pairwise Pearson's correlation coefficients (*r*) of the voxel time courses across the subjects, resulting in 406 unique pairwise *r*‐values for each voxel in each condition, and corrected for multiple comparisons using the threshold‐free cluster enhancement (TFCE) option (Smith & Nichols, 2009). Our main interest lay in finding similarities in brain activity during processing of the same narrative presented via different means. Thus, we additionally performed between‐condition ISC: BOLD signal time courses from each condition (listening, reading, and lipreading) were used as a model to identify brain areas with similar time courses in another condition (Figure 1d). This provided a measure for those brain areas during lipreading that responded similarly to the brain responses measured during listening or reading. Specifically, given a subject pair with one participant in condition *c1* and a second participant in condition *c2*, for each voxel we compute the average ***r*** statistic as the average of all pairwise correlations between the BOLD signal time series *s*(t) at the voxel (formula 1). Unthresholded brain maps for all can be found in Neurovault (/collections/BJRMDQXU/).

(1) $$r_{\mathrm{between}-\mathrm{conditions}\mathrm{ISC}}=1/N_{\mathrm{pairs}}\Sigma_{i=1,\text{…},N;j=1,\text{…},N;i\neq j}{\mathrm{correlations}}_{i,c1}\left(t\right),s_{j,c2}\left(t\right)$$

Due to the fact that the similarity values are not independent (each value depends on two participants), permutation‐based statistical tests were performed. To obtain the statistical significance of the between‐condition ISC and to control for multiple comparisons, whole brain permutations (*N* = 5000) were performed with FSL randomize. With the TFCE option, FSL randomize performs a permutation‐based one sample *t*‐test by flipping the sign of each pairwise correlation to construct surrogate data. TFCE finds “cluster‐like” structures in the data without selecting an a priori cluster‐forming threshold and produces corrected voxel‐level statistics (Smith & Nichols, 2009).

#### 2.3.3 Predicting brain activity with lipreading skills and experienced comprehension

To reveal the brain areas related to lipreading skills and experienced comprehension of the lipread narrative, we predicted the ISC between listening and lipreading against the objective lipreading test score as well as subjective experienced of comprehension. We used the mean of the subjective experienced comprehension rating to mitigate possible moment‐to‐moment differences in the rating of the narrative during and after scanning (Jääskeläinen et al., 2022). For each voxel, the pairwise BOLD similarity between two participants in the two different conditions was modeled with both the objective lipreading test score and the subjective comprehension ratings of the participant in the lipreading condition in each pair. To test for significance of the association between similarities, we ran a Mantel test (Mantel, 1967), which is a non‐parametric test where subjects’ labels are shuffled at each permutation. To obtain statistical significance of the association and to jointly control for multiple comparisons, whole brain permutations (*N* = 5000) were performed with FSL randomize using TFCE. Additionally, to verify that the associations of lipreading comprehension and ISC were not driven by prior exposure to the narrative in the reading or listening conditions, the linear effects of presentation order were regressed out both from the pairwise ISCs and lipreading ratings and the analyses were repeated. To control for the linear effects, we calculated the mean ISCs and mean lipreading scores and ratings for each of the subgroups (i.e., those for who the lipreading condition was first, second, and third, respectively) and subtracted the mean values from each participants’ data before recalculating the correlations. This is equivalent to including the groups as nuisance covariates in a typical GLM analysis employed in fMRI studies. Finally, because of the dependencies between the pairwise ISCs, we repeated the statistical tests with the participants modeled as exchangeability blocks in FSL randomise. Due to the limited sample size and potential limitations of this (Beta stage) feature in accounting for the dependencies in pairwise data, we present the results with both the standard permutations (assuming independence of data) and by modeling the within‐subject dependencies.

## 3 RESULTS

### 3.1 Lipreading skills of participants

**FIGURE 2:**
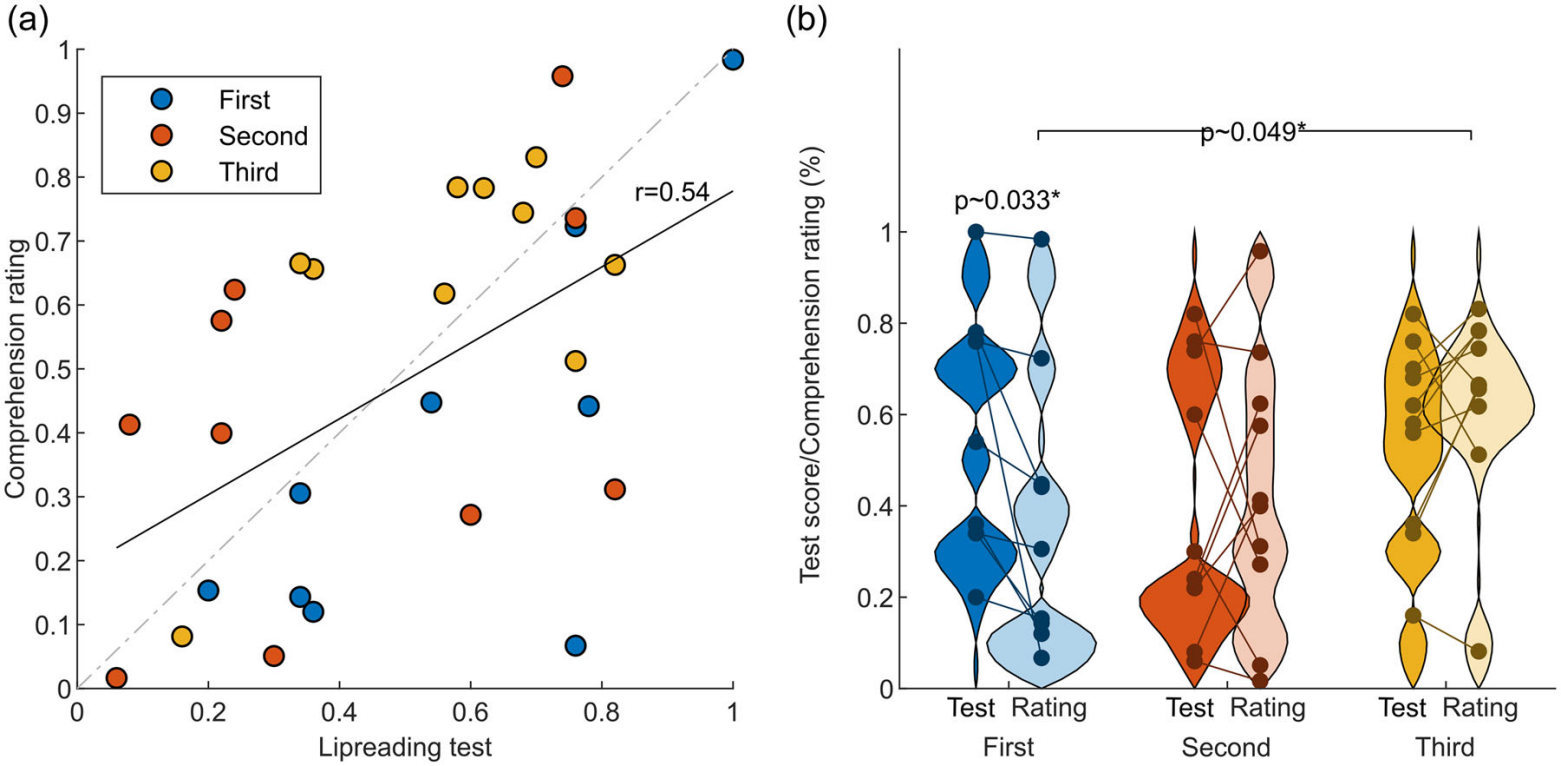
(a) Correlation between lipreading test score and subjective comprehension rating and (b) distribution of test scores and comprehension ratings as a function of presentation order. The ordinal position of the lipreading condition is depicted by the colors of dots and the distributions (blue, lipreading first, red, second, orange, third). In panel B, pale‐colored distributions depict the comprehension ratings while the saturated colors show depict the test score distributions. Individual subjects’ are shown as dots and test and rating data from the same participant are connected by lines

Participants were pre‐screened with a web‐based lipreading test, and prior to the fMRI scanning, their lipreading skills were estimated with a previously validated test, which consisted of 50 words in 10 sentences of variable length (Lonka, 1993). Results of the lipreading test is displayed in Figure 2. Inter‐individual variability was very large with the proportion of correctly recognized words ranging from 6% to 100% (mean = 50.7%; SD = 26). According to the Shapiro–Wilk normality test (*W* = 0.94, *p*‐value = 0.082), the lipreading test results followed a normal distribution. After the fMRI, participants used a dynamic rating tool to provide a continuous subjective estimate of how well they could lipread the stimulus narrative. This estimate was different during different parts of the narrative (Figure 1b). The mean, calculated over the whole narrative, ranged from 0.016 to 0.98 (scale 0−1). Linear regression showed a significant (*r* = 0.54, *p* < 0.003) correlation between lipreading score and mean subjective rating of comprehension (Figure 2a), demonstrating that the lipreading score predicted comprehension of the narrative in the scanner moderately well. Because the presentation order of the different narrative modalities was randomized across participants, some participants listened to and/or read the narrative prior to lipreading. We examined the effect of the presentation order to the subjective ratings (Figure 2b) and found that the participants who had listened to and read the narrative before lipreading (yellow), had slightly better subjective comprehension than participants who lipread narrative first (blue; *p* ∼ 0.049). However, examining the correlation at the level of individual participants (Figure 2b) revealed that the difference was due to significantly poorer subjective rating (*p* ∼ 0.033) compared to lipreading test performance in the group who lipread the narrative first, particularly driven by two participants dropping from almost 80% lipreading test score to ∼40% and ∼20% comprehension rating, respectively (blue) (Figure 2b). Importantly, the correlation between test scores and comprehension ratings persisted after controlling for the presentation order (*r*∼0.58, *p* = 0.0014).

### 3.2 Brain imaging results

#### 3.2.1 Inter‐subject correlation during different narrative types

**FIGURE 3:**
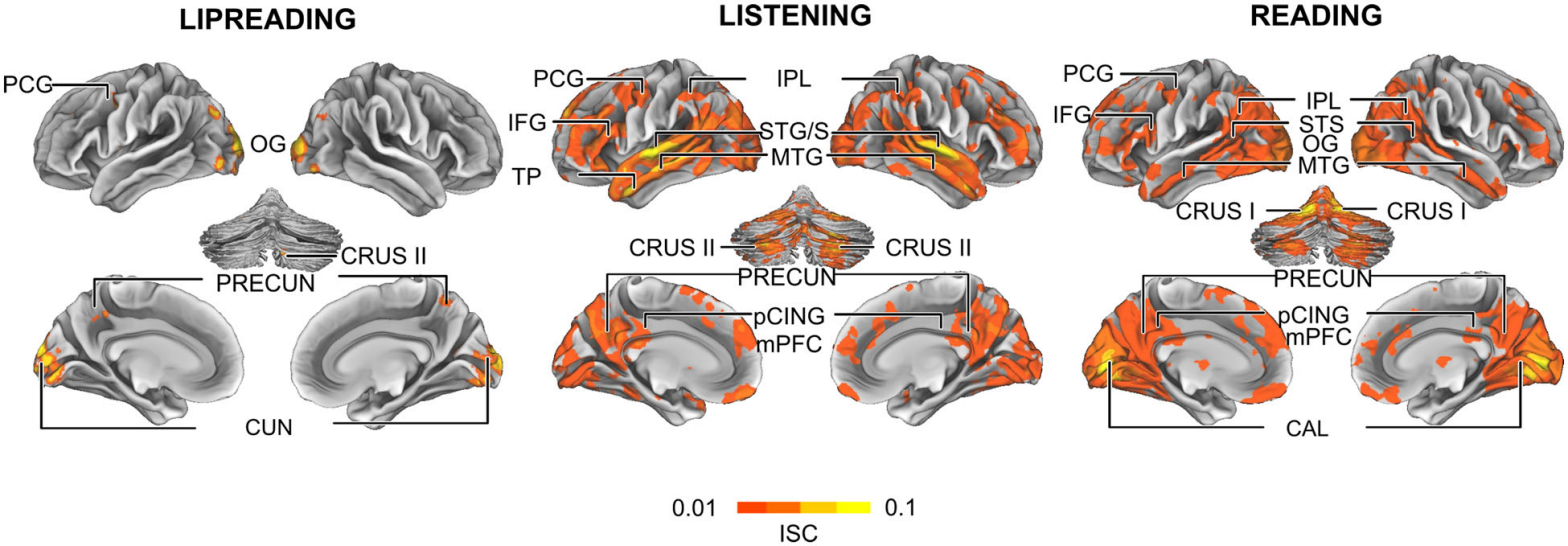
Significant inter‐subject correlation (ISC) during lipreading, listening to, and reading the same narrative (cluster corrected *p* < .05). During lipreading, significant ISC was restricted to visual areas, and a few small clusters were found in the cuneus, precentral gyrus, precuneus and lingual gyri. During listening to and reading the narrative, similarity of activation extended from the temporo‐parietal and frontal language areas to midline. CAL, calcarine sulcus; CRUS I, cerebellar lobule I; CRUS II, cerebellar lobule II; CUN, cuneus; IFG, inferior frontal gyrus; IPL, inferior parietal lobule; M1, primary motor cortex; mPFC, medial prefrontal cortex; MTG, middle temporal gyrus; OG, occipital gyri; pCING, posterior cingulate; PRECUN, precuneus; STG/S, superior temporal gyrus/sulcus; TP, temporal pole

We first computed the ISC of voxel‐wise BOLD time courses to identify shared hemodynamic activity during each of the narrative types. During lipreading (Figure 3, left), significant ISC was mainly found in cortical areas processing different aspects of visual information (the occipital gyri, supracalcarine cortex, cuneus, lingual gyri). Small clusters with significant ISC were also found in the precuneus (PRECUN), precentral gyrus (PCG), and cerebellum (peak values in Table S1).

When the participants listened to the narrative, significant ISC was found bilaterally in the auditory and peri‐auditory cortices, in the superior temporal gyrus and sulcus (STG/S), in the middle temporal gyrus and sulcus (MTG/S), the temporal pole (TP), the IPL as well as the cerebellum (Figure 3 middle, peak values Table S2). In the left hemisphere, significant ISC was found in the superior frontal gyrus (SFG), PCG, and IFG. In the cortical midline, significant ISC was found bilaterally in the PRECUN, pCING, medial prefrontal cortex (mPFC), and visual cortex.

When the participants read the narrative, significant ISC was found bilaterally in visual areas in the cuneus (CUN) and around the calcarine sulcus (CAL), as well as in MTG, IPL, IFG, and CRUS I of the cerebellum. Therefore, the pattern of significant ISCs was rather similar to that during listening, excluding the auditory and periauditory areas, but including more widespread lateral and ventral visual areas and extensive bilateral activity of the occipital midline areas around the CAL instead.

#### 3.2.2 Similarity of brain activity during lipreading, listening to, and reading the narrative measured with between‐condition ISC

Next, we directly examined the similarity of brain activity between lipreading and listening conditions, and between lipreading and reading conditions, meaning that we calculated between‐condition ISCs.

**FIGURE 4:**
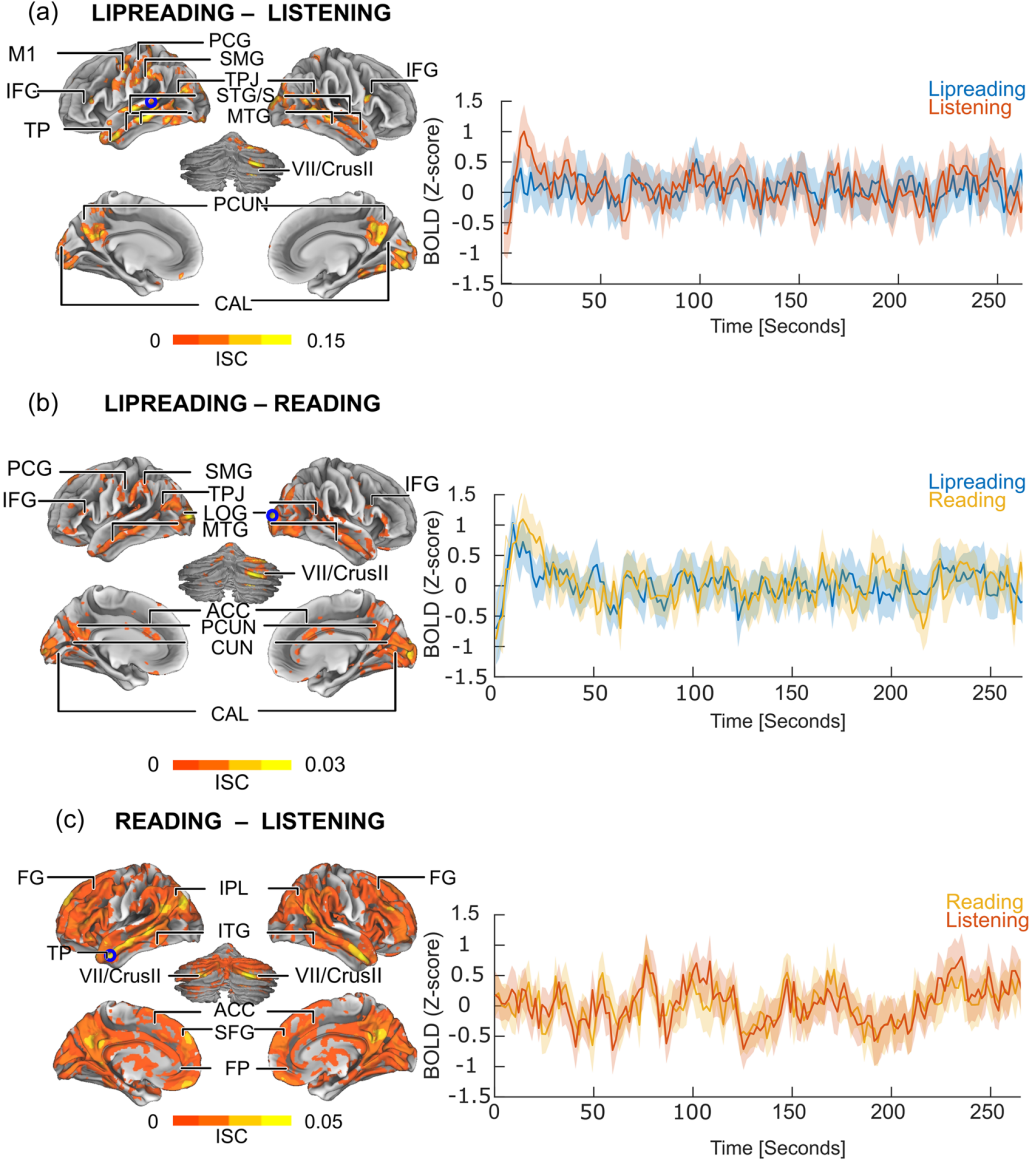
ISC between narrative types. Left: Brain areas showing significant ISC during processing of two different narrative types (permutation‐based cluster correction, *p*_corrected_ < .05). Right: Time series and confidence intervals of peak values of BOLD signal strengths from the largest cluster (denoted with a blue circle) where the signals were significantly similar. (a) During lipreading and listening, maximum similarity (denoted with a circle) was centered at MNI coordinates *x* = −62, *y* = −22, *z* = − 4. (b) During lipreading and reading, maximum similarity was centered at MNI coordinates *x* = 22, *y* = −98, *z* = 12. (c) During listening and reading, maximum similarity was centered at MNI coordinates *x* = −54, *y* = 10, *z* = −30. ACC, anterior cingulate cortex; CAL, calcarine sulcus; Crus II, cerebellar lobule II; CUN, cuneus; FG, inferior frontal gyrus; FP, frontal pole; IPL, inferior parietal lobule; ITG, inferior temporal gyrus; ITP, temporal pole; FP , frontal pole; M1, primary motor cortex; MTG, middle temporal gyrus; LOG, lateral occipital gyrus; PCG, postcentral gyrus; PCUN, precuneus; SFG, superior frontal gyrus; SMG, supramarginal gyrus; STG/S, superior temporal gyrus/sulcus; TPJ, temporo‐parietal junction

ISC between lipreading and listening (Figure 4a, peak values in Table S4, see also Figure S2) was significant in the middle and posterior STG/S, as well as along the whole bilateral MTG/S extending to the temporal pole (TP), left SMG, and right IFG. Furthermore, ISC was significant in two areas of the left primary motor cortex (M1) corresponding approximately to the hand (Yousry et al., 1997) and mouth representation areas (Fox et al., 2001). ISC was also significant in the bilateral PCUN, occipital midline areas around the calcarine sulcus (CAL) (extending to the lingual gyrus [LG] and the right fusiform gyrus [FG]), right‐lateralized areas of the cerebellum, and left somatosensory cortex. By comparison (Figure S3), between‐condition ISC for lipreading and listening to gibberish narratives was significant only in the left middle and posterior STG, and PCG as well as bilateral middle MTG, as well as in the cerebellum (CRUS I and II).

ISC between lipreading and reading was significant (Figure 4b, see also Figure S2 and peak values in Table S5) along the whole MTG/S extending to TP, left SMG, and bilateral lateral occipital gyri (LOG), starting from the occipital pole (OP), and extending around CS, comprising the primary visual cortex as well as other early visual areas. Furthermore, ISC was significant in the left and right cerebellum, PCUN, and cuneus, as well as in the anterior cingulate (ACC). By comparison (Figure S3), between‐condition ISC for lipreading and reading gibberish narratives was significant around the LOG, bilateral IFG, and right posterior MTG.

ISC between reading and listening was significant in an extensive set of brain areas excluding the primary somatosensory cortex, ventrolateral visual cortex, subcortical areas (not shown in the figure), frontal orbital cortex, and right supra‐temporal auditory cortex (Figure 4c; see also Figure S2 and peak values in Table S6). By comparison (Figure S3), between‐condition ISC for reading and listening to gibberish narratives was significant, around the CAL and CUN as well as CRUS I and II in the cerebellum.

**FIGURE 5:**
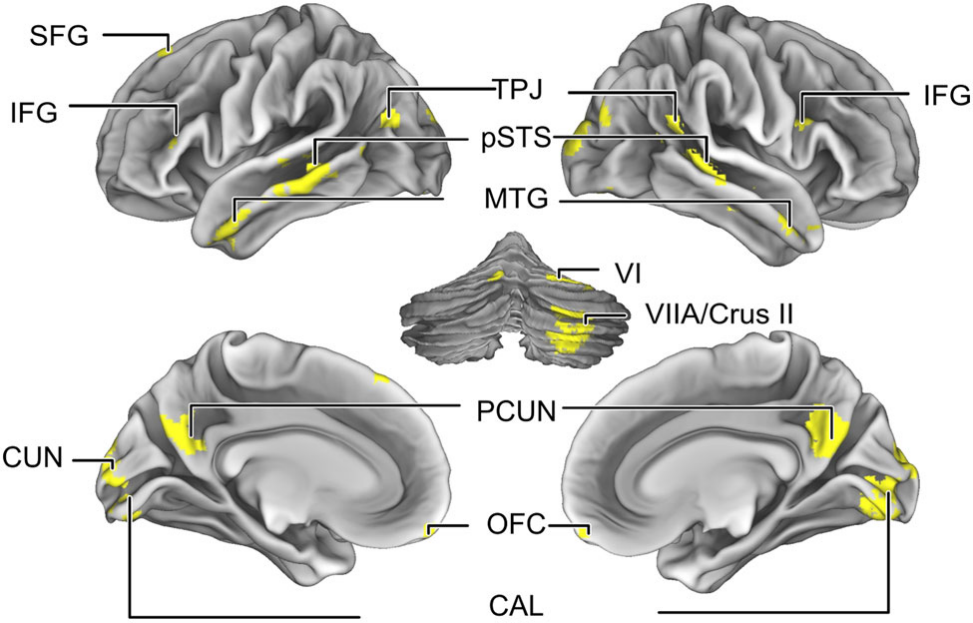
A binary mask indicating brain areas showing overlap for all between‐condition ISCs: areas showing similarity were parts of the fronto‐temporal (SFG, IFG, STG/S, MTG/S), parietal (TPJ), bilateral midline areas (PRECUN, OFC), and right cerebellum (VI and VIIA/Crus II. Crus II, cerebellar lobule II; IFG, inferior frontal gyrus; MTG, middle temporal gyrus; OFC, orbito‐frontal cortex; PCUN, precuneus; SFG, superior frontal gyrus; STS, superior temporal sulcus; TPJ, temporo‐parietal junction

Figure 5 depicts the overlap (conjunction with minimal statistics; Nichols et al., 2005) of the three between‐condition ISCs of Figure 4. These areas comprise the left middle MTG, and SFG, and bilaterally anterior MTG, posterior STS, TPJ, IFG and CS, PCUN, orbitofrontal cortex (OFC), and right cerebellum (VI and VIIA/Crus II).

For intact narratives, the observed neural similarity between lipreading and listening as well as lipreading and reading reflected higher‐order information, although to a lesser extent than between listening and reading. The significant between‐condition ISC of lipreading and listening to gibberish (Figure S3) was restricted to STG and STS, middle MTG and around CAL, CRUS I and II in the cerebellum. Similarly, significant ISC of lipreading and reading gibberish (Figure S3), was restricted to LOG and CUN with only small clusters in the MTG. The significant between‐condition ISC during gibberish narratives did not include PRECUN, anterior MTG, and areas around the posterior STG/S, which were identified to have shared activation during all intact narrative types (Figure 5).

#### 3.2.3 Brain activity predicted by lipreading skills and subjective experienced comprehension

We then identified brain areas where ISC during lipreading and listening covaried with participants’ lipreading skills by estimating how the objective lipreading test score and subjective experienced comprehension predicted the ISC between lipreading and listening (see Figure 4a). We did this by linearly regressing ISC first with the lipreading test score of the participant in each pair, and then the subjective lipreading comprehension rating of the participant in the lipreading condition in each pair. In both cases the data were thresholded with permutation‐based cluster correction (TFCE corrected *p* < .05). Additionally, the mean effects of the presentation order were regressed out from the ratings and ISCs.

**FIGURE 6:**
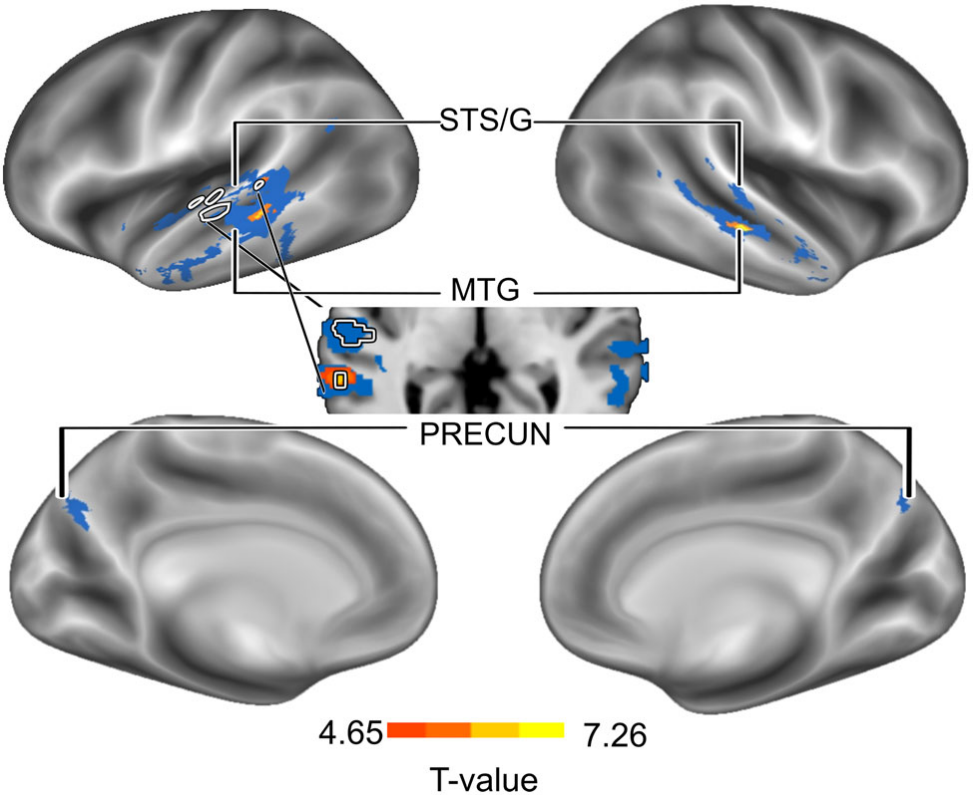
Brain areas showing higher ISC between lipreading and listening in participants with higher subjective rating of comprehension and lipreading test score (white circles). Higher pairwise subjective rating of lipreading comprehension predicted similar brain activity bilaterally in the middle STG/S and MTG when modeling within‐subject dependencies (orange‐yellow). Larger clusters were identified with the standard permutation test extending to bilateral superior and middle temporal areas as well as precuneus (blue), when no dependencies were modeled. Higher pairwise lipreading test score (white circles) predicted similar brain activity in clusters in the left middle and posterior STG/S and in the Sylvian fissure, when modeling within‐subject dependencies. All analyses with permutation‐based TFCE *p*< .05. MTG, middle temporal gyrus; PRECUN, precuneus; STS/G, superior temporal sulcus/gyrus

In a mantel test, the ISC was significantly associated with objective lipreading test score in the middle STG and Sylvian fissure close to the auditory cortex (Figure 6 white circles), and the results were significant in the left hemisphere. Instead, the ISC was significantly associated with experienced comprehension (Figure 6 orange) bilaterally in the middle (peaks at *x* = −60, *y* = −40, *z* = 3; *x* = 62, *y* = −24, *z* = 2) and MTG (peaks at *x* = −53, *y* = −37, *z* = −5; *x* = 54, *y* = −23, *z* = −7), when modeling the within‐subject dependencies in the permutation test. Using the standard permutation test that assumes independent samples (Figure 6 blue), these clusters extended to bilateral middle and posterior STG/S and middle to anterior MTG (not including the primary auditory cortex; see also Figure S2) with an additional cluster in the PRECUN. The superior temporal‐cortical areas showed significant ISC when participants listened to speech, and mid‐temporal cortical areas as well as PRECUN showed significant ISC in all conditions (see Figure 3).

## 4 DISCUSSION

This study has illuminated the neural processes involved in lipreading naturalistic, connected speech in adults with normal hearing and varying lipreading skills by estimating similarity of brain activity during lipreading, listening to, and reading the same narrative. Significant between‐condition ISC demonstrated similar processing of a lipread, listened to, and read narrative in the bilateral anterior MTG, IFG, posterior STS, TPJ, PCUN, as well as areas around Crus II of the right cerebellum (Figure 4). In addition, lipreading and listening to the narrative shared activity in the superior temporal cortex, predominantly in the left STG. Additionally, lipreading and reading activated similar areas in the visual cortex in the occipital lobe. Finally, better comprehension of connected speech via lipreading was associated with increased recruitment of the same temporo‐cortical brain areas during lipreading as those during listening to speech, bilaterally (Figure 6).

### 4.1 Brain areas underlying lipreading, listening to, and reading a narrative

When the participants listened to the narrative (Figure 3), within‐condition ISC was significant bilaterally in the temporo‐parietal (STG/S, MTG/S, IPL), frontal (SFG, PCG, IFG), as well as midline areas (PRECUN, mPFC). Previous research has reliably identified these areas as the extended linguistic network (Fedorenko et al., 2011, bib29; Friederici, 2011; Hickok & Poeppel, 2007; Wilson et al., 2008). When the participants read the narrative (Figure 3), the pattern of significant within‐condition ISC was largely similar to this network, but excluding the superior temporal cortex and including extensive bilateral activity of the visual cortices instead (see also Regev et al., 2013). In contrast to reading and listening, significant within‐condition ISC during lipreading was restricted to visual cortices (Figure 3), with small clusters in the PCG, PRECUN, and lingual gyri most likely due to limited and idiosyncratic comprehension of the narrative. The message was unambiguous in both listening and reading conditions, explaining the strong similarity in brain activity in both within‐ and between‐conditions ISCs. This enabled us to use BOLD time courses from these conditions to model brain activity during lipreading.

Between‐condition ISC (Figure 4) identified lipreading‐related activation in the auditory speech processing pathways in the temporal cortex (Calvert & Campbell, 2003; Pekkola et al., 2005; Sams et al., 1991) and in the multimodal posterior STS/STG (Calvert & Campbell, 2003; Möttönen et al., 2002), also extending to more anterior and lateral areas (Bello et al., 2019). As a control, we performed between‐condition ISC for lipreading and listening to gibberish narratives and it was also significant in these areas (Figure S3), suggesting similar use of the low‐level speech processing areas. However, we did not find significant ISC in the primary auditory cortex in the left hemisphere (Figure 3 and Figure S2), in contrast to previous research using simple stimuli such as syllables and words (Calvert & Campbell, 2003; Pekkola et al., 2005; Sams et al., 1991). We regressed out the responses to auditory and visual transients, such as naturally occurring pauses in the narrative from the BOLD signals, as they are known to trigger activity in the primary sensory and other cortical areas (Lu et al., 2016). It is quite possible that auditory or visual transients, rather than linguistic stimulus features, caused primary auditory‐cortical activation in the above‐mentioned previous studies (Bernstein & Liebenthal, 2014; Bernstein et al., 2011; Paulesu et al., 2003), which may have been removed due to our efforts to remove these effects from the current data. We also found lipreading‐related activation in the anterior parts of the STG and MTG (Figure 4), which have been implicated in mapping acoustic phonetic cues into lexical representations (Binder et al., 2000; Scott et al., 2000). This is in line with the recent suggestion that the STS contains pathways that process facial and vocal signals (Deen et al., 2020). Thus, the same brain areas that are involved in the sound‐based coding of phonemes in the mid‐STG and MTG may underlie processing of visual speech gestures. Lipreading connected speech also appears to be enabled by the convergence of auditory speech pathways (Calvert et al., 1999; Pekkola et al., 2005; Sams et al., 1991).

We also found lipreading‐related activation in the primary motor cortex in the left hemisphere similarly to listening (Figure 3a), but not reading (Figure 3b). In line with previous studies with isolated, simple linguistic stimuli, brain areas related to speech production were activated during lipreading (Callan et al., 2014; Skipper et al., 2005, 2007; Watkins et al., 2003). Apparently, motor knowledge of articulatory gestures modulates auditory‐cortical processing through reciprocal sensory–motor connections (Chu et al., 2013; Kauramäki et al., 2010; Skipper et al., 2005, 2007). Furthermore, motor knowledge of our own speech production is used in lipreading others (Calvert & Campbell, 2003; Nishitani & Hari, 2002; Paulesu et al., 2003; Watkins et al., 2003). This could also be related to the involvement of the somatosensory areas (Möttönen, Järveläinen, Sams, & Hari, 2005). Furthermore, activity in the inferior parietal regions around the temporo‐parietal junction (TPJ; Figure 3a–c) could be related to accessing the stored motor representations of speech during visual speech gesture decoding (Calvert & Campbell, 2003), visuomotor transformations and planning intentions (Fogassi & Luppino, 2005; Shum, Shiller, Baum, & Gracco, 2011; Desmurget, & Sirigu, 2010). There is also evidence of a specific visuomotor pathway involving the middle part of the left MTG and frontoparietal motor areas, mediating speech motor control (O'Sullivan et al., 2017; Venezia et al., 2017). Interestingly, our results also demonstrate that the right cerebellum (cerebellar lobule VI and VII/Cruss II) shows a similar activity during all narrative types (Figure 3). The cerebellum has been found to contribute to motor (production), cognitive (also speech and language), and perceptual functions, and to temporal processing (Kotz & Schwartze, 2010; Kotz et al., 2014). Here, its activity during all narrative types suggests that it is involved in complex linguistic processing and dynamic processing of the narrative stimuli (Buckner, 2013; Kotz et al., 2014; Stoodley et al., 2012; Wang et al., 2013).

We expected the brain areas underlying high‐level linguistic processing during lipreading to be shared between listening and reading because previous research has suggested that they are invariant to modality (Nguyen et al., 2019; Regev et al., 2013). We found that this was true for listening and reading (Figure 4) as they similarly activated brain areas related to high‐level language processing (Booth, Wood, Lu, Houk, & Bitan, 2007; Fairhall & Caramazza, 2013; Fedorenko et al., 2011; Mahowald & Fedorenko, 2016; Regev et al., 2013). Based on the extensive similarity, even cognitive operations such as experiencing emotions, imagery related to the content of the story, social reasoning, and memory (Bar, 2007; Boldt et al., 2013; Fairhall & Caramazza, 2013; Nummenmaa et al., 2014) were most likely involved. However, lipreading connected speech elicited less activation in the higher‐order brain areas (Figure 3 and Figure 4). Lipreading is much more difficult than listening to speech (Altieri et al., 2011; Files et al., 2015), and comprehension of the narrative was lower and less consistent (see also Skipper et al., 2005), leading to less synchronized activity than in the simulation results (Figure S1). Nevertheless, it can be speculated that activity in the bilateral posterior STG and MTG, PCUN, as well as in the left IFG likely reflects processing of higher levels of language, and context (Fairhall & Caramazza, 2013; Wilson et al., 2008), and possibly also mentalizing (Raichle et al., 2001; Simony et al., 2016; Utevsky et al., 2014; Yeshurun et al., 2017b), but more experimental evidence is needed to verify this interpretation. Moreover, some of the brain areas activating similarly to all narrative types (Figure 5) (IPL, lateral temporal cortex, and PRECUN) belong to the default mode network (DMN) (Raichle et al., 2001), which takes part in prospective and episodic memory and self‐referential decision‐making (Buckner & Carroll, 2006; Rugg & Vilberg, 2013). DMN connectivity has been shown to change during narrative listening, perhaps related to accumulating and integrating information over long time scales (Simony et al., 2016).

### 4.2 Brain areas enabling successful comprehension via lipreading

We found temporal‐cortex activity that covaried with objective lipreading score and subjective estimate of lipreading comprehension, suggesting that these cortical areas are most important for lipreading connected speech (Figure 6). The results in respect to different measures of lipreading were in large part aligned, but the effect of comprehension ratings were stronger resulting in larger clusters bilaterally. These results are in line with previous findings on the significant relationship between left STG (Capek et al., 2008; Hall et al., 2005; Ludman et al., 2000) and STS (Blank & Kriegstein, 2013) activation during lipreading isolated sentences and performance in the lipreading test. Our data with naturalistic narrative and subjective experienced comprehension extend these findings, as we also found that lipreading comprehension predicted activity in the right STG, bilateral middle STS, and middle and anterior MTG as well as PRECUN.

Previous studies have identified STG and mid‐posterior STS as the core cortical region for processing acoustic features of speech and in assessing sublexical structures such as phonetic characteristics of words and syllables (DeWitt & Rauschecker, 2012; Hickok & Poeppel, 2007; Liebenthal et al., 2005; Mesgarani et al., 2015). Therefore, these data suggest that lipreading shares bilateral phonological processing pathways with auditory speech (Hickock & Poeppel, 2016). However, there are findings suggesting that the STG is multisensory (Okada et al., 2013), and the set of activated neurons may be different during listening to speech and lipreading even within the same cluster of voxels (Beauchamp et al., 2004). According to Hauswald et al. (2018), auditory regions from the dorsal stream (Hickok, 2012; Hickok & Poeppel, 2007) map the visual speech signal into an absent acoustic signal. Extending this finding, our results suggest lipreading comprehension of connected speech covaries with the activity in the anterior and middle MTG (Figure 6), which reflects subsequent selection and integration of semantic information (Binder et al., 2009; Friederici, 2011; Price, 2010) and lexical‐syntactic information retrieval (Rodd et al., 2015; Snijders et al., 2009). Furthermore, we found that the temporal‐cortex activity related to subjective lipreading comprehension was bilateral (Figure 6). Naturalistic auditory speech stimuli such as narratives consistently elicit more bilateral brain activity than less complex stimuli (Honey et al., 2012; Huth et al., 2016; Jung‐Beeman, 2005; Regev et al., 2013). The activity of the PRECUN, the functional core of the DMN, may be related active processing of the narrative stimulus (Simony et al., 2016).

In summary, we found activity in the superior temporal cortex showing convergence with sublexical auditory speech pathways, and in the middle temporal cortex indicating the involvement of lexical and semantic processing. Furthermore, this activity was associated with both performance in the objective lipreading test as well as subjective experienced comprehension and was, to some extent, activated similarly not only by lipreading, but also by listening to and, to some extent, by reading the narrative. Therefore, these data suggest that modality‐specific visual speech processing does share pathways with auditory speech stimulus processing, and this enables successful lipreading comprehension.

### 4.3 Methodological considerations

It is important to note that ISC is a tool that builds on stimulus processing‐evoked activity so that brain areas where such extrinsic activity is prominent and similar in participants (e.g., visual‐cortex activity during viewing a feature film) show strong ISC. But brain activity is always the sum of extrinsic and intrinsic activity, where intrinsic activity refers to all brain activity in the same brain area which is not related to the experimental manipulation. The fact that we found just a few areas with significant within‐condition ISC during lipreading but extensive areas showing significant between‐condition ISC indicates that lipreading‐triggered brain activity was not strong or consistent enough to serve as a model for other participants’ brain activity (see Figure S1 for simulation). However, as also indicated in Figure 4, listening to and reading the narrative activated the brain substantially more strongly, providing such a model for studying similar processing across narrative types, increasing the sensitivity of our analyses. Potentially, pattern analyses leveraging multivariate spatial patterns, such as inter‐subject functional connectivity (Simony et al., 2016) across conditions, could reveal further information on the differences and similarities of functional networks participating in language comprehension in different modalities.

Further, the presentation sequence of three narrative types were randomized across participants and some participants listened to and/or read the narrative prior to lipreading. Therefore, for some participants, previous listening to the narrative might have influenced neural processing during reading or lipreading the narrative. This might have, due to adaptation and familiarity, also influenced the ISC between listening and reading, which, however, was extensive. In addition, reading or listening to the narrative before lipreading might have made lipreading easier for some participants. Although controlling for the presentation order does not significantly alter the results in the current manuscript, it is something to consider in future studies. In addition, using only one speaker might have modified the corresponding neural processing due to familiarization (Sanchez et al., 2013).

It has also been previously shown that voxel‐by‐voxel cross‐subject comparisons are complicated because of considerable anatomical variability, which is especially true for areas that are sensitive to low‐level visual speech features (Bernstein et al., 2011). These issues should be resolved in further research, which could study a set of good lipreaders several times each to calculate ISCs. This could also illuminate the possibly idiosyncratic processing strategies of skilled lipreaders, and would enable reaching higher than moderate correlation between the lipreading test and subjective estimation of comprehension. Furthermore, a study including male participants is needed in order to verify the generalizability of results across sexes (Ruytjens et al., 2006, 2007), as more attention would be drawn to the possible differences in lipreading processing between females and males (Bernstein, 2018). In the current study, we did not coerce eye gaze, but rather expected and allowed free focus shifts between the mouth and the central area of the face and eyes during lipreading (Paré et al., 2003), because previous studies with hearing adults have suggested that individuals’ ability to lipread silent spoken sentences (Lansing & McConkie, 2003) and consonant–vowel–consonant clusters (Wilson, Alsius, Paré, & Munhall, 2016) did not correlate with mouth focus. However, the effect of direct mouth gaze on lipreading should be considered more carefully in future studies.

## 5 Conclusions

Our results show that in addition to activity in visual, auditory, and speech motor areas, lipreading a naturalistic narrative activated higher‐level modality‐independent brain areas similarly to listening to or reading the same narrative. However, activation during lipreading was much more limited than with listening and reading, probably mirroring the more limited comprehension during lipreading. Importantly, better comprehension of lipread connected speech was enabled by bilateral activity in the superior and middle temporal gyri and sulci, suggesting an overlap in neural processes that are involved in auditory coding of phonetic speech features.

## AUTHOR CONTRIBUTIONS

SS: Planned the experiment, prepared stimuli, collected and analyzed data, prepared figures, wrote the manuscript as first author. JA: Planned the experiment, collected and analyzed data, wrote the manuscript. JML: Planned the experiment, prepared and edited stimuli, analyzed data, wrote the manuscript. MB‐T: Planned the experiment, wrote the manuscript. EG: Developed methods, analyzed data, wrote the manuscript. IPJ: Planned the experiment, supervised collecting data, wrote the manuscript. UH: Planned the experiment, wrote the manuscript. MS: Principal investigator of the study, planned and supervised the experiment, wrote the manuscript.

## CONFLICT OF INTEREST

The authors declare that there is no conflict of interest, and that they have no competing financial interests.

### PEER REVIEW

The peer review history for this article is available at https://publons.com/publon/10.1002/brb3.2869.

## Supporting information

Figure S1. Simulation of three scenarios where 29 subjects are studied in three different conditions (color coded with green, violet, and grey) with different levels of ISC strengths

Figure S2. Inter‐subject correlation between narrative types with primary auditory cortex outlines (smaller circle) based on Jülich 2 mm probabilistic anatomical maps {Formatting citation}

Figure S3. ISC between gibberish narrative types

Table S1. Peak values of inter‐subject correlation (ISC) of lipreading to Figure 3.
Table S2. Peak values of inter‐subject correlation (ISC) of listening to Figure 3
Table S3. Peak values of inter‐subject correlation (ISC) of reading related to Figure 3
Table S4. Peak values of between condition ISC: lipreading and listening
Table S5. Peak values of between condition ISC: lipreading and reading
Table S6. Peak activations of between condition ISC: listening and reading

## Data Availability

The data that support the findings of this study are available on request from the corresponding author SS. The data are not publicly available due to restrictions, for example, their containing information that could compromise the privacy of research participants.
