# Supplementary figures and images for: Lipreading a naturalistic narrative in a female population: Neural characteristics shared with listening and reading
Source: Brain Behav. 2022 Dec 29;13(2):e2869. doi: 10.1002/brb3.2869 (PMC9927859; doi:10.1002/brb3.2869)

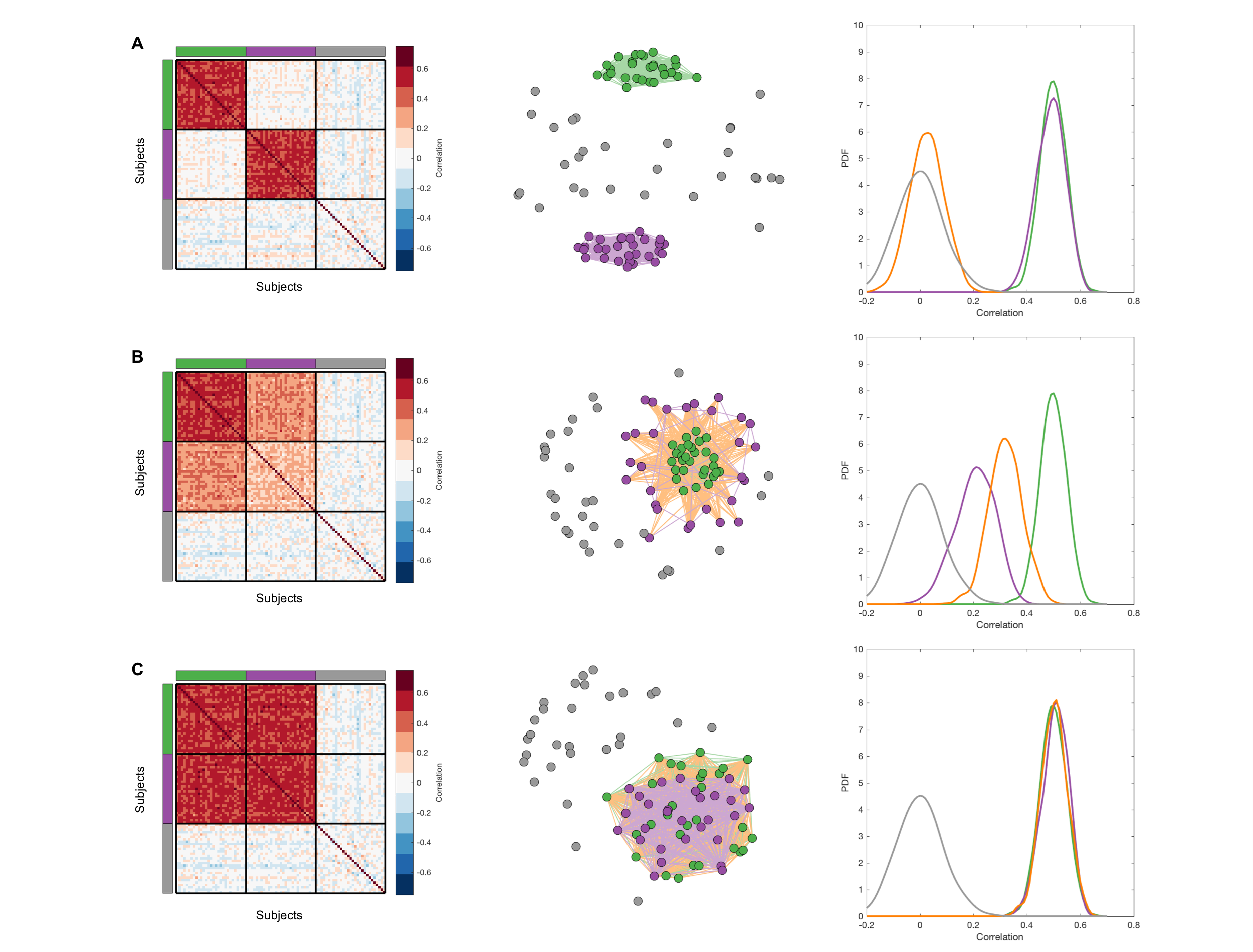

Supplement: Supplementary file 1 — Figure S1. Simulation of three scenarios where 29 subjects are studied in three different conditions (color coded with green, violet, and grey) with different levels of ISC strengths [file BRB3-13-e2869-s003.tif]

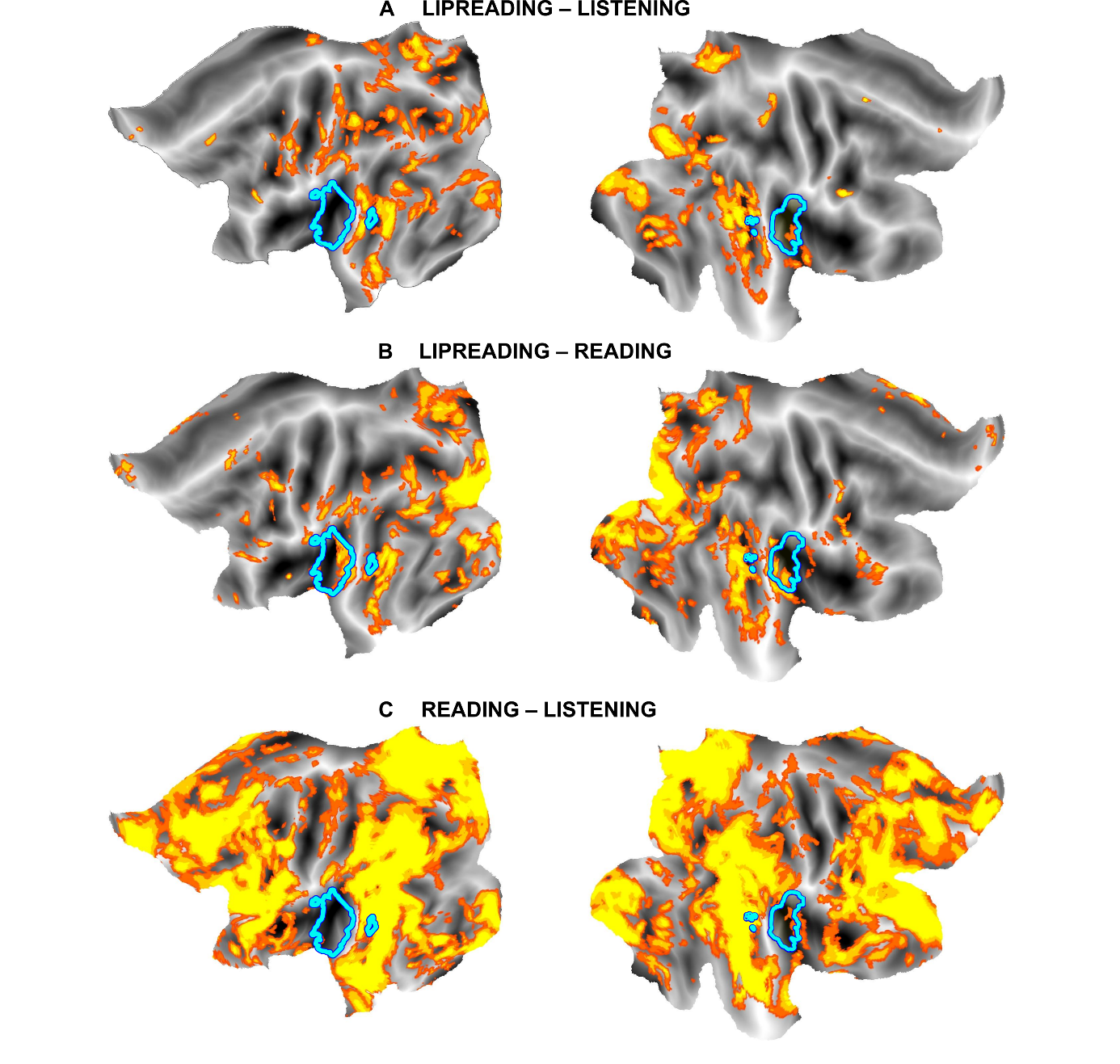

Supplement: Supplementary file 2 — Figure S2. Inter‐subject correlation between narrative types with primary auditory cortex outlines (smaller circle) based on Jülich 2 mm probabilistic anatomical maps {Formatting citation} [file BRB3-13-e2869-s002.tif]

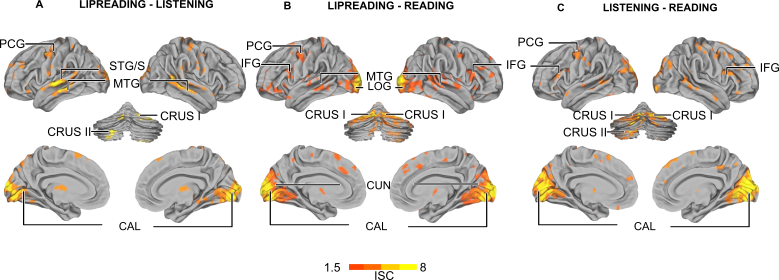

Supplement: Supplementary file 3 — Figure S3. ISC between gibberish narrative types [file BRB3-13-e2869-s004.tif]
