# Supplementary material for: Lipreading a naturalistic narrative in a female population: Neural characteristics shared with listening and reading
Source: Brain Behav. 2022 Dec 29;13(2):e2869. doi: 10.1002/brb3.2869 (PMC9927859; doi:10.1002/brb3.2869)
Supplement: Supplementary file 4 — Table S1. Peak values of inter‐subject correlation (ISC) of lipreading to Figure 3. Table S2. Peak values of inter‐subject correlation (ISC) of listening to Figure 3 Table S3. Peak values of inter‐subject correlation (ISC) of reading related to Figure 3 Table S4. Peak values of between condition ISC: lipreading and listening Table S5. Peak values of between condition ISC: lipreading and reading Table S6. Peak activations of between condition ISC: listening and reading [file BRB3-13-e2869-s001.docx]

**Lipreading a naturalistic narrative in a female population: neural characteristics shared with listening and reading**

Saalasti, S.^1,2,3*^, Alho, J.^1^, Lahnakoski, J.M.^1,4,5,6^, Bacha-Trams, M.^1^, Glerean, E.^1,7^, Jääskeläinen, I.P.^1^, Hasson, U.^7^ & Sams, M.^1,8^

1 Brain and Mind Laboratory, Department of Neuroscience and Biomedical Engineering, Aalto University School of Science

2 Department of Psychology and Logopedics, University of Helsinki

3 Advanced Magnetic Imaging (AMI) Centre, Aalto NeuroImaging, School of Science, Aalto University, Espoo, Finland

4 Independent Max Planck Research Group for Social Neuroscience, Max Planck Institute of Psychiatry, Munich, Germany

5 Institute of Neuroscience and Medicine, Brain & Behaviour (INM‐7), Research Center Jülich, Jülich, Germany

6 Institute of Systems Neuroscience, Medical Faculty, Heinrich Heine University Düsseldorf, Düsseldorf, Germany

### 7 Department of Psychology and the Neuroscience Institute, Princeton University

### 8 Department of Computer Science, Aalto University School of Science

Correspondence*: satu.saalasti@helsinki.fi

Supplemental Information

**Table S1. Peak values of inter-subject correlation (ISC) of lipreading to Figure 3. Reported p-values are FDR corrected.**

| **Cluster ID (hemisphere)** | **Size** | **x** | **y** | **z** | **max value** | **p-value** | |  |
| --- | --- | --- | --- | --- | --- | --- | --- | --- |
| Cuneus (R)[CUN(R)] | 2286 | 16 | -96 | 16 | 0.028226 | | 0.0000022991 | |
| Precentral gyrus (L)[PreCG(L)] | 78 | -48 | 4 | 44 | 0.014551 | | 0.0017685 | |
| Middle occipital gyrus (L)[MOG(L)] | 73 | -38 | -78 | 38 | 0.014856 | | 0.0014198 | |
| Precuneus (R)[PCUN(R)] | 72 | 6 | -56 | 56 | 0.018088 | | 0.0001017 | |
| Inferior occipital gyrus (R)[IOG(R)] | 71 | 42 | -86 | -10 | 0.016894 | | 0.00050493 | |
| Precuneus (L)[PCUN(L)] | 66 | -6 | -52 | 40 | 0.016953 | | 0.00049565 | |
| Calcarine gyrus (R)[CAL(R)] | 53 | 12 | -60 | 20 | 0.013826 | | 0.0024876 | |
| Cerebellar crus II (R)[XII(R)] | 51 | 12 | -80 | -42 | 0.014872 | | 0.0014142 | |
| Middle occipital gyrus (L)[MOG(L)] | 49 | -42 | -86 | -4 | 0.016085 | | 0.00076958 | |
| Superior frontal gyrus (L)[SFGdor(L)] | 41 | -28 | 40 | 38 | 0.014126 | | 0.0021059 | |
| Olfactory cortex (R)[OLF(R)] | 39 | 14 | 10 | -12 | 0.014915 | | 0.0013448 | |
| Lingual gyrus (L)[LING(L)] | 37 | -20 | -80 | -14 | 0.014662 | | 0.0016805 | |
| Superior temporal gyrus (R)[STG(R)] | 37 | 54 | -22 | 16 | 0.012738 | | 0.0040354 | |
| Lingual gyrus (L)[LING(L)] | 33 | -28 | -98 | -12 | 0.013946 | | 0.0024118 | |
| Rolandic operculum (L)[ROL(L)] | 32 | -50 | 0 | 10 | 0.013654 | | 0.0025773 | |
| Middle occipital gyrus (R)[MOG(R)] | 30 | 46 | -74 | 34 | 0.012875 | | 0.0037706 | |

**Table S2. Peak values of inter-subject correlation (ISC) of listening to Figure 3. Reported p-values are FDR corrected.**

| **Cluster ID (hemisphere)** | **Size** | **x** | **y** | **z** | **max value** | **p-value** |
| --- | --- | --- | --- | --- | --- | --- |
| Superior temporal gyrus (R)[STG(R)] | 47710 | 60 | -12 | 0 | 0.090405 | 1.41E-06 |
| Orbital superior frontal gyrus (R)[ORBsup(R)] | 3790 | 34 | 66 | -6 | 0.02947 | 6.33E-06 |
| Gyrus rectus (L)[REC(L)] | 478 | -4 | 54 | -20 | 0.033971 | 6.33E-06 |
| Fusiform gyrus (L)[FFG(L)] | 186 | -30 | -40 | -16 | 0.021341 | 6.33E-06 |
| Amygdala (R)[AMYG(R)] | 152 | 22 | -8 | -16 | 0.017513 | 8.96E-06 |
| Amygdala (L)[AMYG(L)] | 136 | -20 | -8 | -18 | 0.018351 | 7.04E-06 |
| Middle frontal gyrus (L)[MFG(L)] | 97 | -24 | 10 | 56 | 0.017395 | 9.20E-06 |
| Insula (R)[INS(R)] | 87 | 32 | 22 | -6 | 0.013949 | 0.00021078 |
| Cerebellar lobule IX (L)[IX(L)] | 75 | -6 | -54 | -46 | 0.016495 | 2.95E-05 |
| Supplementary motor area (R)[SMA(R)] | 67 | 10 | 16 | 64 | 0.016212 | 3.01E-05 |
| Caudate (R)[CAU(R)] | 61 | 10 | 14 | 6 | 0.01407 | 0.00019436 |
| Caudate (L)[CAU(L)] | 56 | -12 | 0 | 14 | 0.012542 | 0.00069886 |
| Postcentral gyrus (R)[PoCG(R)] | 39 | 28 | -38 | 52 | 0.015097 | 5.76E-05 |
| Orbital medial frontal gyrus (R)[ORBsupmed(R)] | 38 | 2 | 40 | -8 | 0.014718 | 8.73E-05 |
| Rolandic operculum (R)[ROL(R)] | 36 | 54 | 10 | 2 | 0.013838 | 0.00022575 |
| Supramarginal gyrus (L)[SMG(L)] | 32 | -56 | -36 | 34 | 0.014043 | 0.00019682 |
| Postcentral gyrus (R)[PoCG(R)] | 32 | 34 | -32 | 70 | 0.012984 | 0.00051107 |
| Triangular inferior frontal gyrus (R)[IFGtriang(R)] | 31 | 58 | 30 | 8 | 0.013724 | 0.00025832 |
| Putamen (R)[PUT(R)] | 30 | 24 | 8 | 2 | 0.013773 | 0.00024968 |
| Superior frontal gyrus (R)[SFGdor(R)] | 30 | 16 | 56 | 34 | 0.013341 | 0.00038532 |
| Precuneus (R)[PCUN(R)] | 30 | 8 | -52 | 64 | 0.014012 | 0.00019859 |

**Table S3. Peak values of inter-subject correlation (ISC) of reading related to Figure 3. Reported p-values are FDR corrected.**

| **Cluster ID (hemisphere)** | **Size** | **x** | **y** | **z** | **max value** | **p-value** |
| --- | --- | --- | --- | --- | --- | --- |
| Calcarine gyrus (R)[CAL(R)] | 56460 | 12 | -84 | 2 | 0.19706 | 6.14E-07 |
| Medial superior frontal gyrus (L)[SFGmed(L)] | 4774 | -8 | 58 | 28 | 0.036468 | 3.29E-06 |
| Middle frontal gyrus (R)[MFG(R)] | 3150 | 46 | 48 | 4 | 0.030272 | 3.29E-06 |
| Thalamus (R)[THA(R)] | 143 | 0 | -4 | 4 | 0.018771 | 3.29E-06 |
| Superior temporal gyrus (L)[STG(L)] | 137 | -48 | -22 | 6 | 0.015418 | 4.35E-05 |
| Insula (R)[INS(R)] | 80 | 34 | 22 | 2 | 0.018428 | 3.93E-06 |
| Cerebellar crus I (L)[XI(L)] | 71 | -52 | -64 | -42 | 0.014745 | 7.10E-05 |
| Thalamus (L)[THA(L)] | 70 | -4 | -34 | 6 | 0.015263 | 4.51E-05 |
| Caudate (R)[CAU(R)] | 69 | 8 | 10 | 10 | 0.015717 | 2.97E-05 |
| Middle frontal gyrus (L)[MFG(L)] | 61 | -26 | 36 | 32 | 0.016734 | 1.49E-05 |
| Anterior cingulum (L)[ACG(L)] | 45 | -8 | 44 | 0 | 0.014268 | 0.00011345 |
| Amygdala (L)[AMYG(L)] | 42 | -20 | -6 | -18 | 0.014852 | 6.54E-05 |
| Postcentral gyrus (L)[PoCG(L)] | 38 | -26 | -32 | 72 | 0.012659 | 0.00047422 |
| Amygdala (R)[AMYG(R)] | 34 | 16 | -6 | -18 | 0.014429 | 0.00010511 |
| Middle temporal gyrus (L)[MTG(L)] | 31 | -44 | -16 | -8 | 0.015434 | 4.34E-05 |
| Medial superior frontal gyrus (R)[SFGmed(R)] | 30 | 14 | 66 | 16 | 0.013496 | 0.00024803 |
| Thalamus (L)[THA(L)] | 29 | -8 | -4 | 16 | 0.014808 | 6.83E-05 |
| Brainstem [BST] | 28 | 2 | -30 | -4 | 0.013832 | 0.00019304 |
| Anterior cingulum (L)[ACG(L)] | 28 | -2 | 0 | 26 | 0.014594 | 9.17E-05 |
| Postcentral gyrus (R)[PoCG(R)] | 28 | 16 | -38 | 80 | 0.018201 | 4.68E-06 |
| Temporal pole (superior) (R)[TPOsup(R)] | 27 | 32 | 14 | -24 | 0.013402 | 0.00026761 |
| Brainstem [BST] | 28 | 2 | -30 | -4 | 0.013832 | 0.00019304 |

**Table S4. Peak values of between condition ISC: lipreading and listening. Related to Figure 4A. Reported p-values are cluster corrected p-values estimated with FSL randomise TFCE. We used p_corrected < 0.05 as threshold for the table.**

| Cluster ID (hemisphere) | Size | x | y | z | max value | p-value |
| --- | --- | --- | --- | --- | --- | --- |
| Posterior Superior temporal gyrus/sulcus (L)pSTG/S | 16051 | -62 | -22 | -4 | 7.36 | 0.0002 |
| 0Cerebellum VII (R)Cereb VII/CrussII | 303 | 28 | -84 | -40 | 6.8 | 0.004 |
| Superior Parietal Lobule (L)SPL | 176 | 43 | -44 | 68 | 3.81 | 0.0158 |
| Intra-parietal sulcus IPS | 115 | 32 | -60 | 34 | 4.58 | 0.009 |
| Cerebellum (R)Cereb | 111 | 20 | -60 | -58 | 4.9 | 0.0064 |
| Inferior Frontal Gyrus. pars opercularis (R)IFG | 100 | 48 | 14 | 20 | 4.9 | 0.0054 |
| Temporal pole (L)TP | 83 | -44 | 32 | 12 | 4.04 | 0.0162 |
| Middle Frontal Gyrus (L)MFG | 50 | -36 | 2 | 56 | 3.38 | 0.0324 |
| Cerebellum VII/Crus II (R)VII/Crus II | 40 | 36 | -66 | -52 | 3.5 | 0.0322 |
| Precentral gyrus (R)PCG | 38 | 54 | 8 | 40 | 3.52 | 0.0332 |
| Frontal Pole (L)FP | 35 | -16 | 52 | 28 | 3.83 | 0.0318 |
|  |  |  |  |  |  |  |

**Table S5. Peak values of between condition ISC: lipreading and reading. Related to Figure 4B. Reported p-values are cluster corrected p-values estimated with FSL randomise TFCE. We used p_corrected < 0.05 as threshold for the table.**

| Cluster ID | Size | x | y | z | max value | p-value |
| --- | --- | --- | --- | --- | --- | --- |
| Occipital Pole (R)OP | 20760 | 22 | -98 | 12 | 11.2 | 0.0002 |
| Planum Temporale (L)PT | 2519 | -42 | -32 | 14 | 5.35 | 0.0006 |
| Frontal Pole (L)FP | 726 | -50 | 40 | 10 | 4.53 | 0.004 |
| Anterior Cingulate (R)ACC | 353 | 2 | 22 | 22 | 4.86 | 0.0022 |
| Inferior Frontal Gyrus pars opercularis (R)IFG | 224 | 48 | 14 | 26 | 4.65 | 0.0068 |
| Cerebellum VII/CrusII (L)VII/CrussII | 127 | -22 | -76 | -36 | 4.02 | 0.0182 |
| Precentral Gyrus (R)PCG | 105 | 50 | 2 | 44 | 4.35 | 0.0132 |
| Orbitofrontal cortex (L)OFC | 95 | -6 | 30 | -28 | 4.02 | 0.0186 |
| Medial Prefrontal Cortex (L)MPFC | 59 | -10 | 40 | 50 | 3.32 | 0.0322 |
| Anterior Cingulate (R)ACC | 59 | 2 | -2 | 28 | 3.94 | 0.0184 |
| Lateral Occipital Cortex(L)LOC | 58 | -52 | -66 | 42 | 2.46 | 0.0394 |
| Medial Frontal Gyrus (L)MFG | 46 | 8 | 42 | -20 | 3.27 | 0.0322 |
| Medial superior frontal gyrus (L)SFGmed | 43 | -6 | 24 | 66 | 2.95 | 0.044 |
| Middle Frontal Gyrus (L)MFG | 41 | -36 | 6 | 36 | 3.12 | 0.0338 |
| Superior Parietal Lobule (R)SPL | 40 | 26 | -40 | 36 | 2.97 | 0.033 |
| Anterior Cingulate (L)ACC | 29 | -4 | 16 | 36 | 2.78 | 0.0424 |
| Cerebellum VII/Crus II (L)VII/CrusII | 28 | -38 | -70 | -34 | 5.09 | 0.0126 |

**Table S6. Peak activations of between condition ISC: listening and reading. Related to**

**Figure 4C. Reported p-values are cluster corrected p-values estimated with FSL randomise TFCE. We used p_corrected < 0.05 as threshold for the table.**

| Cluster ID | Size | x | | y | | z | max value | p-value |
| --- | --- | --- | --- | --- | --- | --- | --- | --- |
| Temporal pole (L)TP(L) | 109006 | | -54 | | 10 | 30 | 22.1 | 0.0002 |


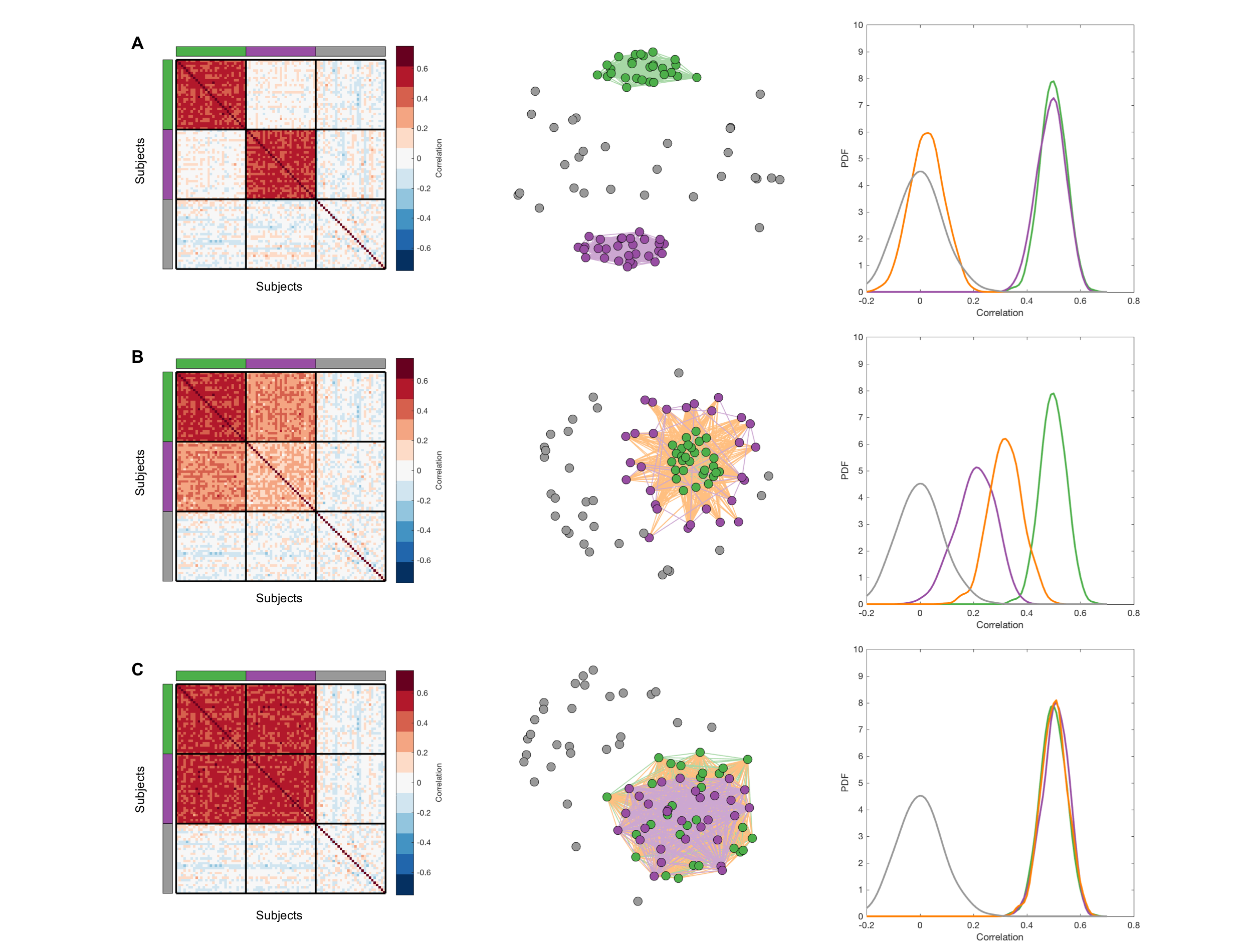


**Figure S1. Simulation of three scenarios where 29 subjects are studied in three different conditions (colour coded with green, violet and grey) with different levels of ISC strengths.** *Left:* ISC matrixes where each element of the matrix shows the correlation between a subject pair. Two subjects might be in the same condition (square matrices along the main diagonal) or in two different conditions (matrices outside the main diagonal). *Middle*: ISC matrix can be visualised as a network where the pairwise correlations are the "link weights" in the network with each node being a subject in one of the three conditions (showing only link weights with r > 0.3 for visualization purposes). *Right*: The distributions of the link weights reflecting inter-subject correlations. The stronger are the correlations the more the distribution is shifted to the right.

**A)** Condition-specific activity timecourses. *Left:* Within-condition ISCs are strong in conditions *green* and *violet*, but non-existent in condition grey. Between-condition ISCs are weak. *Middle:* The subjects in conditions green and violet cluster together, reflecting strong ISC (the closer the nodes, the stronger the pairwise correlations). *Right:* The distributions for within conditions are shifted to the right while the distributions for the correlations between condition green and condition violet (depicted in orange) are comparable to the null distribution (in grey).

**B)** Condition-generic activity timecourse, different signal-to-noise ratios. *Left:* Conditions green and violet share an identical signal, but the signal-to-nose ratio (SNR) is lower in condition violet and, therefore the within-condition ISC of violet condition is weak. However, the between condition ISC is stronger. *Middle:* The subjects cluster as networks, with the green nodes being close to each other, whereas subjects in condition violet are closer to the green but further away from other violets, depicting that the between-condition ISC is stronger than within-condition ISC. This is similar to the case of the listening and lip-reading between condition ISC. *Right:* The distributions of the ISC scores show that the between conditions ISC (orange) is shifted towards the right more than the violet, as it has stronger correlations.

**C)** Condition-generic activity timecourse, similar signal-to-noise ratios. *Left:* Conditions green and violet are sharing the same signal which has the same SNR across conditions, resulting in strong within condition ISC and between condition ISC. This is similar to the case of listening and reading between condition ISC. *Middle*: The subjects in condition green and violet cluster together so that clusters of each colour are close to one-another. *Right:* The distributions of the ISC scores show that the between conditions ISC are equally shifted to the right reflecting equal strength of correlations.


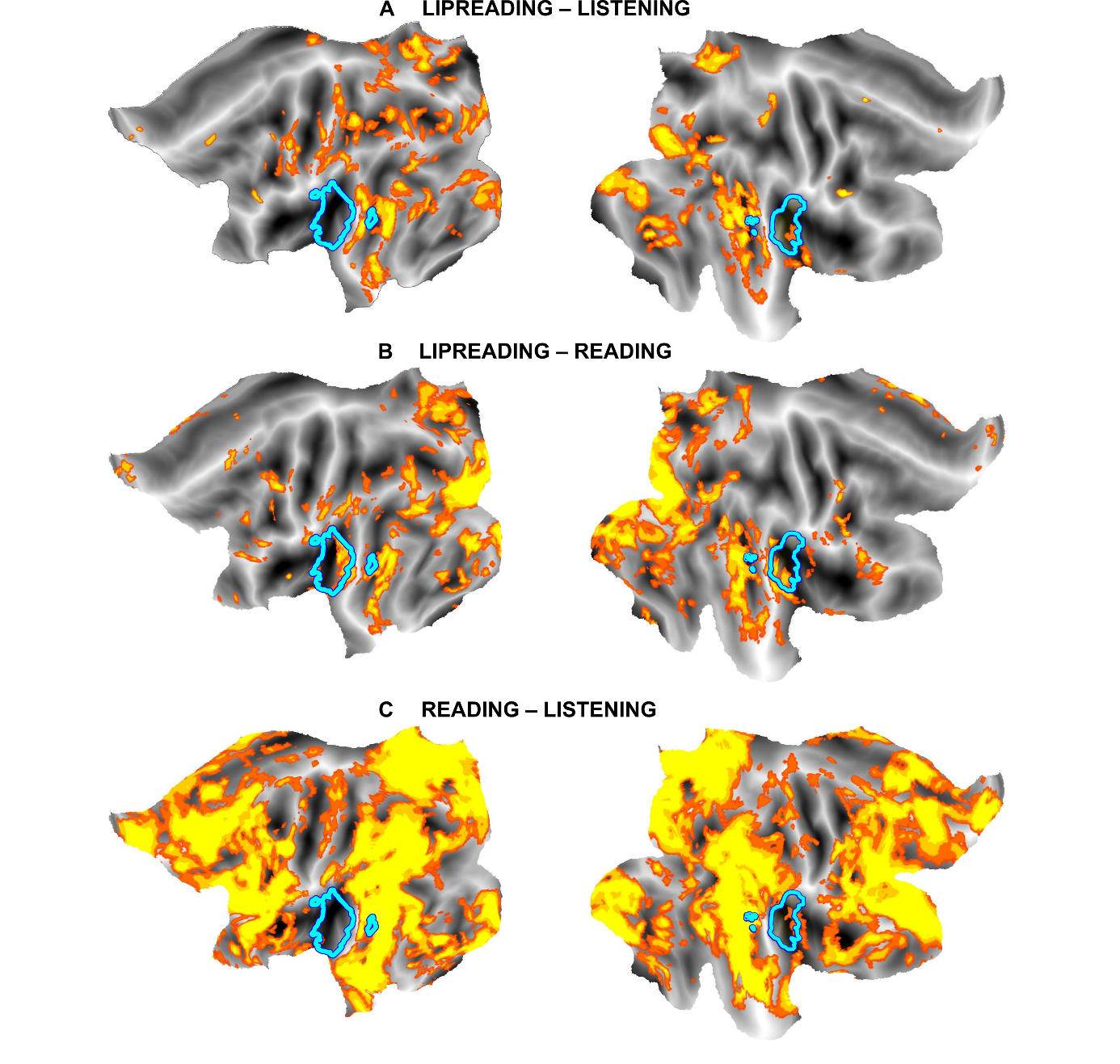


**Figure S2. Inter-subject correlation between narrative types with primary auditory cortex outlines (smaller circle) based on Jülich 2mm probabilistic anatomical maps** **{****Formatting itation}.**

**
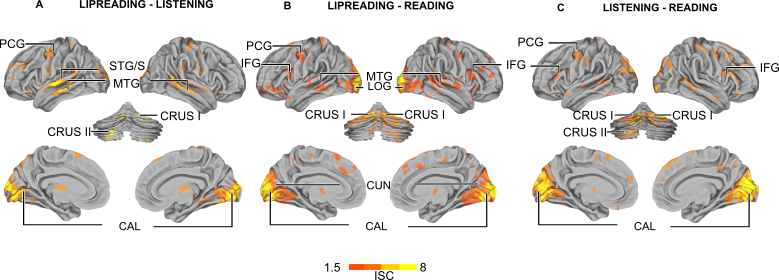
**

**Figure S3. ISC between gibberish narrative types. Brain areas showing significant ISC during processing of two different gibberish narrative types** (permutation-based cluster-correction, p*_corrected_* < 0.05)**.** A) During lipreading and listening to gibberish, significant ISC was restricted to STG and STS and middle MTG and around CAL, as well as CRUS I and II in the cerebellum. B) During lipreading and reading gibberish, similarity was restricted to LOG and CUN with only smaller clusters MTG C) During listening to and reading gibberish, significant ISC was restricted to CAL and CUN as well as CRUS I and II in the cerebellum

**Stimulus narrative translated to English.**

I woke up to the persistent sound of my alarm clock. Waking up felt heavy, but I forced myself out of bed. I turned off the nasty sound of my alarm clock and stretched for a moment. Half asleep I made my bed and pulled a blouse and trousers on me. After opening the curtains, I felt considerably more awake. Light that was rushing in told me that spring had advanced. I turned around and headed towards the bedroom door. On my way, my foot hit a sauna bucket which was lying behind the bed and made a ruckus when falling. I had to curse for a moment when the pain hit my toes, but then I lifted the bucket up and headed towards the kitchen.

I went straight to the fridge. Jar of yoghurt and pear were an adequate breakfast. For some reason, my husband Jarkko’s mobile phone was also in the refrigerator. I was beginning to be in a hurry for work, but I nonetheless brewed some coffee, the aroma of which floated delightfully into my nose. Suddenly I felt hands begin to rub my shoulders. Jarkko had appeared behind me and he managed to surprise me pleasantly. When I wondered why Jarkko had not yet left for work, he replied that he had felt nauseous during the small hours. He was going to go right back to rest.

While sipping the last of my breakfast coffee, I gave the weary Jarkko a kiss. I pulled a jacket on me, I put my shoes on and stepped outside. As the door opened, beautiful birdsong filled the air. Gravel rattled underneath my shoes as I hurried to the car. Before jumping behind the wheel, I noticed Jarkko's backpack on the roof of my car. So typical, I smiled to myself, as I carried the backpack back inside. When I left it on the floor in the hallway, I noticed Jarkko quickly stopping a phone call and blushing almost as if guilty. However, Jarkko assured that someone had just called the wrong number. I was in a hurry so I asked no further questions, but hasted to my car.

Engine ran steadily, as I drove through the peaceful rural landscape. The terrain varied with forests and fields. On ridges grew pines and in valleys dense spruce. In other places the road crossed over small rapids. Nature already started to turn green, much to the influence of the spring sun.

I reflected on the behaviour of Jarkko this morning: his sudden disconnection of the phone call and blushing as if guilty. I wonder whether Jarkko had something inappropriate going on with someone. Would he guess that he had awakened my doubts, and, if so, would he be scared enough to terminate the relationship. I wondered if I would dare to spy from Jarkko’s phone who the caller was if I had the opportunity? Would Jarkko suspect that and empty his phone-call records? Maybe I should confront Jarkko if the call data had been cleared.

I woke to the reality from these gloomy reflections, when a large tree fell with a crash across the road. Brakes screeched as I struggled to stop the car before a crash! I climbed out of the car to see what had downed the tree. To my surprise, I saw a brown-haired, square teethed creature at the foot of the tree, and the root of the tree had bite marks. Beaver! When I approached the beaver, he fled deeper into the forest. Although we had already lived for about five years in Canada, this was the first time I saw a beaver. Shaking my head, I hastened back to the car, I turned the car around and I planned an alternate route to work in my mind. At the same time I took the phone from my pocket and I called the local emergency number, to declare fallen timber.

As I entered the elevator in the parking garage of my working place, I came across James, a handsome man with whom I had had a secret romance in the fall. James had begun to suspect that his wife knows something, and then we agreed that we take a break and let things cool down. I suspected, however, that James was just tired of me, and that his wife had actually not suspected anything. It may well be that James had another lover, and he did not want to mix things up too much. But I hid my doubts and I chit chatted with him. James and I were going to the same meeting. On the way to a meeting room, a chain-saw that someone had left in the corridor caught my attention.

The meeting proved to be a tight match. The aim was to agree on delivery of some goods, but I doubted from the very beginning the other party's ability to deliver on the schedule and at the price required. During the meeting, the salesmen began to get nervous, when they realized that I was not ready to swallow their excuses so easily. In the end, it was only decided that we will get back to the matter after giving it some thought overnight.

There was a surprise waiting for me when I stepped out of the office. A crowd of colleagues had gathered in the coffee room and they congratulated me for my five-year time tenure at the firm. I was given a chainsaw as a gift! The very same one, which I had wondered about in the corridor. Sure, the people at work knew that I lived in a rural area and a chainsaw would be of use. At the same time, I doubted, though, that there was a bit of sarcasm included. During my time as a CEO I had cut more than one less lucrative branch of the company off.
